# Semiparametric accelerated failure time models with time-varying covariates under partly interval censoring

**DOI:** 10.1186/s12874-026-02916-y

**Published:** 2026-06-13

**Authors:** Aishwarya Bhaskaran, Ding Ma, Benoit Liquet, Angela Hong, Stephane Heritier, Serigne N. Lo, Jun Ma

**Affiliations:** 1 School of Mathematical and Physical Sciences, Macquarie University, Sydney, NSW Australia; 2 School of Mathematics and Statistics, University of New South Wales, Sydney, NSW Australia; 3 The University of Queensland’s Clinical Trials Capability (ULTRA) Team, Brisbane, QLD Australia; 4 Laboratoire de Mathématiques et de leurs Applications, Université de Pau et des Pays de l’Adour, E2S UPPA, CNRS, Anglet, France; 5 Melanoma Institute Australia, The University of Sydney, Sydney, NSW Australia; 6 Faculty of Health and Medicine, The University of Sydney, Sydney, NSW Australia; 7 Department of Radiation Oncology, Chris O’Brien Lifehouse, Sydney, NSW Australia; 8 GenesisCare, Radiation Oncology, Mater Sydney Hospital, Sydney, NSW Australia; 9 School of Public Health and Preventive Medicine, Monash University, Melbourne, VIC Australia; 10 Charles Perkins Centre, The University of Sydney, Sydney, NSW Australia

## Abstract

In survival analysis, accelerated failure time (AFT) models are often used as an alternative to the Cox model. A benefit of the AFT model is that it provides a direct link between covariates and event times, and therefore offers meaningful interpretations of the regression coefficients directly on the event times. This paper is motivated by a randomised clinical trial dataset on advanced melanoma patients, in which the event times are interval-censored and one of the covariates in the model is time-varying. To allow direct assessment of the effects of covariates on the event time, we aim to fit an AFT model. However, there are no readily available computational packages for AFT models with time-varying covariates and interval censoring. In this paper, we propose a maximum penalised likelihood approach for fitting such models. The effectiveness of our method is demonstrated through extensive simulations. Also, an application to the above mentioned melanoma dataset is used to demonstrate practical utility of our method.

**Supplementary Information:**

The online version contains supplementary material available at 10.1186/s12874-026-02916-y.

## Introduction

The Cox proportional hazards model [1] is the leading semiparametric approach for analysing covariate effects in survival analysis. However, the proportional hazards assumption of the Cox model may not hold in practical applications. In such cases, an alternative is the semiparametric accelerated failure time (AFT) model [2], in which the error distribution is unspecified and therefore treated nonparametrically. Another motivation for using the AFT model is that it provides a direct relationship between the covariates and the event time.

This paper was motivated by the WBRTMel trial, a multinational, open-label, phase III randomised controlled trial that compared whole brain radiotherapy (WBRT) with the observation (or the control) group. Patients with 1 to 3 melanoma brain metastases, treated initially with surgical excision and/or stereotactic irradiation, were randomised to either the WBRT arm or the control arm. The primary outcome is distant intracranial failure within 12 months of randomisation, assessed by magnetic resonance imaging (MRI) performed every 3 months over the 12-month follow-up period. In the original analysis, this endpoint was treated as a binary outcome (intracranial failure within 12 months: yes/no). The relative treatment effect was summarised using the odds ratio and its 95% confidence interval, estimated from a logistic regression model without covariate adjustment, as specified in the statistical analysis plan [3, 4]. In this paper, we will re-analyse this dataset using a semiparametric survival regression model.

To conduct a survival analysis using this dataset, we first had to define the event time of interest. The failure time was defined as the time from randomisation to distant intracranial progression. Patients without evidence of intracranial progression by the end of follow-up were right-censored. For patients who experienced progression, the exact event was not directly observed but occurred at scheduled MRI assessments. Therefore, the event time was interval-censored, with the interval defined as the time between the last scan showing no progression and the first scan confirming progression. In cases where progression was detected at the first post-baseline scan, the event was treated as left-censored. Another feature of this study was that some patients received systemic therapy, which was time-dependent, during the study period. Given the efficacy of systemic therapy in extending time to progression [5], it is crucial to account for this time-varying covariate when assessing the effectiveness of WBRT.

Clinicians were interested in assessing the direct effects of covariates on the time to distant intracranial failure and therefore preferred to use an AFT model, in which both time-fixed and time-varying covariates were present and the event times were subject to interval censoring. However, there is currently a lack of readily available statistical software capable of handling AFT models with both time-varying covariates and partly interval-censored data.

Motivated by the need to handle interval censoring, and more generally partly interval censoring [6], in the presence of time-varying covariates, as well as to develop corresponding computational tools, this paper proposes an efficient method for fitting semiparametric AFT models under such settings.

Several estimation methods for semiparametric AFT models, but only with time-fixed covariates, exist in the literature, including rank-based approaches [2, 7, 8], the Buckley-James estimator and its generalisations [9–11], smoothed error distribution approximations [12], and baseline hazard approximations [13]. However, extending most of these methods to settings involving both time-varying covariates and interval censoring remains a substantial challenge.

Time-varying covariates are covariates whose values change over time. In biomedical research, common examples include biomarkers and cumulative treatment exposures, typically recorded at scheduled or random intermittent time points prior to the event time or right-censoring time. To handle time-varying covariates within the semiparametric AFT framework, Zeng and Lin [14] proposed a kernel-smoothed profile likelihood approach to enable gradient-based estimation; however, their method is restricted to right-censored data. Another estimation method for semiparametric AFT models with time-varying covariates, also limited to right-censored survival times, is provided in Ma et al. [15].

Partly interval-censored survival data, as defined by Kim [6], include exact event times as well as left-, right-, and interval-censored observations. For example, in studies of chronic illnesses, event status is typically assessed at periodic follow-up visits, naturally leading to interval censoring. Given the prevalence of interval censoring in practice, it is important to account for such data in survival regression models. A commonly used strategy of replacing each censoring interval with a single imputed value, such as the midpoint, can introduce severe bias in regression estimates (e.g., Ma et al. [16]). While the idea of multiple imputation, as proposed by Pan [17] for Cox models, can be applied to AFT models, it becomes computationally prohibitive due to the need for repeated model fitting.

In this paper, we propose a maximum penalised likelihood method for fitting the semiparametric AFT model with time-varying covariates, where event times are partly interval-censored. In this approach, the nonparametric baseline hazard is approximated using basis functions, and the selected penalty function helps (i) obtain a smoothed baseline hazard estimate and (ii) mitigate the impact of the number and placement of knots used to define the basis functions.

Although our approach shares similarities with the framework of Ma et al. [15], it is more than a simple extension. While Ma et al. [15] considers AFT models with time-varying covariates under right-censoring, accommodating interval censoring introduces substantial additional complexity. Unlike right-censored data, where likelihood contributions correspond to single time points, interval-censored data require computing the probability that an event occurs within a given interval, resulting in more complicated likelihood expressions and potential numerical instabilities, especially when the censoring intervals are narrow.

Furthermore, our work is closely related to Webb and Ma [18], who consider Cox models with time-varying covariates under interval censoring. However, the modelling framework is fundamentally different. In the Cox model, the baseline hazard is a function of time, whereas in the AFT setting it is expressed as a function of a transformed time scale that depends on both time-fixed and time-varying covariates. This induces a nonlinear, parameter-dependent transformation that fundamentally alters the structure of the likelihood and the associated estimation problem.

The remainder of this paper is organised as follows. In the Notation section, we introduce the notation required for this paper. The Semiparametric AFT model with time-varying covariates section formally presents the model definition for a semiparametric AFT model with time-varying covariates and partly interval censoring. In the Maximum penalised likelihood estimation section, we describe the maximum penalised likelihood estimation procedure for estimating the regression coefficients and baseline hazard, along with the development of asymptotic results that accommodate active constraints. The results from the simulation studies and the real data application are presented in the Simulation and Application sections, respectively. Finally, we conclude with the Discussion section by discussing the relevance of the new method and outlining potential directions for future research.

## Notation

Let $T_i$ denote the failure time for individual *i*, where $1 \le i \le n$. Under a partly interval censoring scheme, each individual is associated with a random interval denoted by $\boldsymbol{Y}_i = [Y_i^L, Y_i^R]^T$, such that $T_i \in [Y_i^L, Y_i^R]$ with $Y_i^L \le Y_i^R$. The observed interval is denoted by $\boldsymbol{y}_i = [y_i^L, y_i^R]^T$. Now let $\delta _i$ indicate an observed failure time and let $\delta _i^L$, $\delta _i^R$, and $\delta _i^I$ indicate left, right, and interval censoring respectively. Accordingly, each $\boldsymbol{y}_i$ corresponds to either an event ($y_i^L = y_i^R = t_i$, $\delta _i = 1$), left censoring ($y_i^L = 0$, $\delta _i^L = 1$), right censoring ($y_i^R = \infty$, $\delta _i^R = 1$), or interval censoring ($0\ne y_i^L < y_i^R \ne \infty$, $\delta _i^I = 1$).

Each individual has both time-fixed covariates $\boldsymbol{x}_i = (x_{i1}, \dots , x_{ip})$ and time-varying covariates $\boldsymbol{z}_i(t) = (z_{i1}(t), \dots , z_{iq}(t))$ at any time *t*. Let $\tilde{\boldsymbol{z}}_i(t) = \{\boldsymbol{z}_i(s): s \le t\}$ denote the history of the time-varying covariates up to time *t*. Note that in practice, the time-varying covariates $\boldsymbol{z}_i(t)$ are typically observed at discrete time points $t_{i1}, \dots , t_{in_i}$ under an intermittent sampling scheme, with $t_{i1} = 0$. This indicates that different individuals may have distinct sampling schedules and different length $n_i$ for their time-varying covariates. Without loss of generality, we assume that if $T_i$ is right-censored, then $t_{in_i} = y_i^L$; otherwise, $t_{in_i} = y_i^R$.

Therefore, the observed data consist of $(\boldsymbol{y}_i, \delta _i, \delta _i^L, \delta _i^R, \delta _i^I, \boldsymbol{x}_i, \tilde{\boldsymbol{z}}_i(y_i^*))$, where $y_i^* = y_i^LI(y_i^R=\infty )+y_i^RI(y_i^R\ne \infty )$ [18] and *I(· )* denotes the indicator function. Throughout this article, we also assume independent censoring given the covariates; see, for example, Chapter 1 of Sun [19].

## Semiparametric AFT model with time-varying covariates

Following Cox and Oakes [20], the AFT hazard model formulation with time-varying covariates can be written as

1 $$\begin{aligned} \lambda (t \mid \boldsymbol{x}_i,\tilde{\boldsymbol{z}}_i(t)) = \lambda _0(\kappa _i(t;\boldsymbol{\beta },\boldsymbol{\gamma }))e^{-\boldsymbol{x}_i\boldsymbol{\beta }-\boldsymbol{z}_i(t)\boldsymbol{\gamma }}, \end{aligned}$$

with

2 $$\begin{aligned} \kappa _i(t;\boldsymbol{\beta },\boldsymbol{\gamma }) = e^{-\boldsymbol{x}_i\boldsymbol{\beta }}\int ^t_0e^{-\boldsymbol{z}_i(s)\boldsymbol{\gamma }}ds , \end{aligned}$$

where $\boldsymbol{\beta }$ and $\boldsymbol{\gamma }$ are *p × 1* and *q × 1* vectors of regression coefficients for the time-fixed and time-varying covariates respectively.

In the AFT literature, $\lambda _0(\kappa (t))$ is referred to as the baseline hazard function as it represents the instantaneous risk when all covariates are set to zero. Unlike the Cox model framework, where the baseline hazard is expressed as a function of time, the AFT formulation involves a transformed time scale $\kappa _i(t;\boldsymbol{\beta },\boldsymbol{\gamma })$ that depends on the regression parameters. As a consequence, the regression parameters enter both through the hazard component and through the transformed time scale, resulting in a more involved likelihood structure and a more challenging estimation problem. Note that in the semiparametric AFT model, $\lambda _0(\kappa (t))$ is unspecified. The proposed formulation incorporates the full history of the time-varying covariate process $z_i(t)$ through the quantity $\kappa _i(t;\boldsymbol{\beta },\boldsymbol{\gamma })$. In particular, the integral term accumulates the contribution of $z_i(s)$ over the interval *[0, t]*, so that the likelihood depends on the entire observed trajectory of the time-varying covariate up to time *t*, rather than only its value at the observed failure or censoring time. In this work, the process $z_i(t)$ is treated as an observed external time-dependent covariate process, and inference is conducted conditional on its observed trajectory. That is, we do not specify a joint longitudinal-survival model for $z_i(t)$.

For notational convenience, we adopt the $\kappa _i$ notation in (1) for the purpose of simplifying notations throughout this paper. Furthermore, we write $\lambda _0(\kappa (t))$ as $\lambda _0(\kappa )$ when no ambiguity arises.

The cumulative hazard associated with (1) is

$$\begin{aligned} \Lambda (t \mid \boldsymbol{x}_i,\tilde{\boldsymbol{z}}_i(t)) = \Lambda _0(\kappa _i(t;\boldsymbol{\beta },\boldsymbol{\gamma })), \end{aligned}$$

where $\Lambda _0(\kappa (t)) = \int ^{\kappa (t)}_0\lambda _0(s)ds$ is the cumulative baseline hazard. The corresponding survival function can then be expressed as

$$\begin{aligned} S(t\mid \boldsymbol{x}_i, \tilde{\boldsymbol{z}}_i(t)) = S_0(\kappa _i(t;\boldsymbol{\beta },\boldsymbol{\gamma })), \end{aligned}$$

with $S_0(\kappa (t))= \exp \{-\Lambda _0(\kappa (t))\}$. To simplify the notation in this article, we will henceforth denote $\lambda (t \mid \boldsymbol{x}_i,\tilde{\boldsymbol{z}}_i(t))$ and $\Lambda (t \mid \boldsymbol{x}_i,\tilde{\boldsymbol{z}}_i(t))$ by $\lambda _i(t)$ and $\Lambda _i(t)$ respectively.

Note that the nonparametric baseline hazard $\lambda _0(\kappa )$ is an infinite-dimensional parameter. Thus, attempting to estimate $\lambda _0(\kappa )$ without any restrictions is an ill-conditioned problem as there only exists a finite number of observations. By adopting the method-of-sieves (e.g. Geman and Hwang [21]), we make use of a sum of *m* basis functions, where *m<<n* but *m* can vary with *n*, to formulate an approximation to $\lambda _0(\kappa )$ as follows

3 $$\begin{aligned} \lambda _0(\kappa ) = \sum \limits _{u=1}^{m}\theta _u\psi _u(\kappa ), \end{aligned}$$

where $\theta _u$ is an unknown but fixed coefficient for each basis function $\psi _u(\kappa )$, for $1 \le u \le m$. Usually we select $\psi _u(\kappa ) \ge 0$ so that the constraint $\lambda _0(\kappa ) \ge 0$ is satisfied by simply imposing $\theta _u \ge 0$ for all *u*. Given that $\Psi _u(\kappa ) = \int _{0}^{\kappa } \psi _u(s) ds$, the cumulative baseline hazard can be expressed as: $\Lambda _0(\kappa ) = \sum _{u=1}^{m} \theta _u \Psi _u(\kappa ).$ Since *κ* depends on $\boldsymbol{\beta }$ and $\boldsymbol{\gamma }$, its boundary is unknown and may change as the estimates of $\boldsymbol{\beta }$ and $\boldsymbol{\gamma }$ are updated during the iterations. This can cause numerical instabilities when estimating $\boldsymbol{\beta }$ and $\boldsymbol{\gamma }$ [15].

The maximum penalised likelihood method of this paper utilises a gradient-based algorithm to find the optimal solution, which requires the existence of the first two derivatives of $\lambda _0(\kappa )$. Consequently, the selected basis functions $\psi _u(\kappa )$ are also required to have first and second derivatives. Many commonly used basis functions can satisfy this requirement, including the Gaussian bases adopted in Ma et al. [15]. One benefit with the Gaussian bases is that they do not have boundaries, which implies that there is no need to update the basis boundaries when $\boldsymbol{\beta }$ and $\boldsymbol{\gamma }$ values change during iterations, thereby improving numerical stability. Other basis functions, such as M-splines, are also possible, but they require special numerical stability procedures. The results for general basis functions for the semiparametric AFT model will be reported elsewhere.

## Maximum penalised likelihood estimation

### Penalised likelihood

Let $\boldsymbol{\theta }$ be an *m × 1* vector for the coefficients of the basis functions. Using the hazard formulation of the semiparametric AFT model shown in (1), the log-likelihood for the observations $(\boldsymbol{y}_i, \delta _i, \delta _i^L, \delta _i^R, \delta _i^I, \boldsymbol{x}_i, \tilde{\boldsymbol{z}}_i(y_i^*))$, $1 \le i \le n$, is as follows:

4 $$\begin{aligned} \ell (\boldsymbol{\beta },\boldsymbol{\gamma }, \boldsymbol{\theta })= & \sum \limits ^n_{i=1}\left( \delta _i\{\log \lambda _i(y_i) -\Lambda _i(y_i)\} + \delta _i^R\{-\Lambda _i(y_i^L)\} + \delta _i^L\log \{1-S_i(y_i^R)\}\right. \nonumber \\ & +\left. \delta _i^I\log \{S_i(y_i^L)-S_i(y_i^R)\}\right) . \end{aligned}$$

The above likelihood formulation accommodates exact, left-, right-, and interval-censored observations within a unified framework, and reduces to the corresponding special cases by setting the relevant censoring indicators $(\delta _i, \delta _i^L, \delta _i^R, \delta _i^I)$ to zero. In particular, when only exact and right-censored observations are present, the likelihood reduces to the formulation considered in Ma et al. [15].

Note that with right-censored data, each subject’s contribution to the likelihood involves either a density $f_i(y_i)$ or a survival function $S_i(y_i)$. In contrast, the likelihood structure with interval censoring is substantially more complex, introducing terms such as $\log \{S_i(y_i^L) - S_i(y_i^R)\}$, which represent the log-probability that an event occurred within a time interval. These expressions make the log-likelihood more challenging to evaluate and differentiate. As a result, the gradient and Hessian structures become considerably more intricate. This added algebraic and computational burden makes inference under interval censoring markedly more demanding than in the right-censored case, necessitating tailored methods such as the one detailed in the remainder of this section.

We adopt a penalised log-likelihood to estimate $\boldsymbol{\beta }$, $\boldsymbol{\gamma }$, and $\boldsymbol{\theta }$. In particular, we use a roughness penalty function for $\boldsymbol{\theta }$. This penalty not only introduces smoothness, it also mitigates the dependence of the estimates on the number and placement of knots required by the approximate baseline hazard function. Hence, greater flexibility and stability in the estimation of the baseline hazard is achieved. Our experience suggests that the penalty function is a crucial component for ensuring numerical stability.

The roughness penalty adopted here is a continuous, derivative-based penalty that directly penalises the curvature of $\lambda _0(\kappa )$, and is naturally suited to the smooth Gaussian basis functions employed in (3). An alternative formulation would be a discrete penalty based on finite differences of neighbouring basis coefficients, as commonly used in P-spline settings [22]. While such a discrete penalty could also be used within our framework, the continuous roughness penalty is preferred here as it is directly tied to the second-derivative structure of the basis functions and provides a natural measure of functional smoothness.

Combining (4) with a roughness penalty (e.g. Eubank [23]), the penalised log-likelihood can be written as

$$\begin{aligned} P(\boldsymbol{\beta }, \boldsymbol{\gamma }, \boldsymbol{\theta }) = \ell (\boldsymbol{\beta }, \boldsymbol{\gamma }, \boldsymbol{\theta }) - h\boldsymbol{\theta }^T\boldsymbol{R}\boldsymbol{\theta }, \end{aligned}$$

where *h \ge 0* is the smoothing parameter and each (*u*, *v*)-th element of matrix $\boldsymbol{R}$ is given by $\int ^{d_2}_{d_1}\psi _u''(s)\psi _v''(s)ds$, with $d_1$ and $d_2$ denoting the boundary values of *κ (t)*. Note that $\boldsymbol{R}$ is fixed once $d_1$ and $d_2$ are fixed.

Thus, the maximum likelihood estimates of $(\boldsymbol{\beta }, \boldsymbol{\gamma }, \boldsymbol{\theta })$ can be obtained by solving the following constrained optimisation problem:

5 $$\begin{aligned} (\hat{\boldsymbol{\beta }}, \hat{\boldsymbol{\gamma }}, \hat{\boldsymbol{\theta }}) = \underset{\boldsymbol{\beta }, \boldsymbol{\gamma }, \boldsymbol{\theta }}{\text {argmax}} P(\boldsymbol{\beta }, \boldsymbol{\gamma }, \boldsymbol{\theta }), \end{aligned}$$

where $\boldsymbol{\theta } \ge 0$, with the inequality interpreted elementwise.

For implementation, we follow standard penalised spline practice: the number of basis functions *m* is allowed to scale with the sample size, and knots are placed at empirical quantiles of the transformed time scale $\kappa _i$; see the Simulation section for details.

### Computational framework

To obtain the constrained solution to (5), we adopt the Karush-Kuhn-Tucker (KKT) necessary conditions and solve them by alternating between pseudo-Newton updates for $\boldsymbol{\beta }$ and $\boldsymbol{\gamma }$ and a multiplicative iterative (MI) algorithm (e.g. Chan and Ma [24]) for $\boldsymbol{\theta }$ within each iteration. The MI algorithm can impose the $\boldsymbol{\theta }\ge 0$ constraint efficiently. Common to basis functions-based smoothing methods, care must be exercised when selecting the number and location of knots in order to maintain numerical stability, especially when intervals are narrow or survival probabilities approach the extremes.

At each iteration, the pseudo-Newton updates are performed using the intricate derivative structures induced by interval censoring. Specifically, for the pseudo-Newton methods, negative definite terms are extracted from the Hessians to construct pseudo-Hessians. These pseudo-Hessians are then used to update $\boldsymbol{\beta }$ and $\boldsymbol{\gamma }$, ensuring that the search direction is uphill. Subsequently, the MI algorithm is applied to update each $\theta _u$, with $\boldsymbol{\beta }$ and $\boldsymbol{\gamma }$ fixed at their current values. The MI algorithm ensures that all $\theta _u$ remain non-negative. This process is repeated for multiple iterations until convergence is achieved.

The procedure is described in more detail below. Note that the analytic expressions of the gradient vectors $\nabla _{\boldsymbol{\beta }}P(\boldsymbol{\beta }, \boldsymbol{\gamma }, \boldsymbol{\theta })$, $\nabla _{\boldsymbol{\gamma }}P(\boldsymbol{\beta }, \boldsymbol{\gamma }, \boldsymbol{\theta })$, and $\nabla _{\boldsymbol{\theta }}P(\boldsymbol{\beta }, \boldsymbol{\gamma }, \boldsymbol{\theta })$ and the pseudo-Hessian matrices $H^{-}_{\boldsymbol{\beta }}P(\boldsymbol{\beta }, \boldsymbol{\gamma }, \boldsymbol{\theta })$ and $H^{-}_{\boldsymbol{\gamma }}P(\boldsymbol{\beta }, \boldsymbol{\gamma }, \boldsymbol{\theta })$ are provided in the supplementary material.

To perform the alternating pseudo-Newton updates within each iteration, we make use of the following equations:

$$\begin{aligned} \boldsymbol{\beta }^{(k+1)}= & \boldsymbol{\beta }^{(k)} - a_{\boldsymbol{\beta }}^{(k)}\left\{ H^{-}_{\boldsymbol{\beta }}P(\boldsymbol{\beta }^{(k)}, \boldsymbol{\gamma }^{(k)}, \boldsymbol{\theta }^{(k)})\right\} ^{-1}\nabla _{\boldsymbol{\beta }}P(\boldsymbol{\beta }^{(k)}, \boldsymbol{\gamma }^{(k)}, \boldsymbol{\theta }^{(k)}), \\ \boldsymbol{\gamma }^{(k+1)}= & \boldsymbol{\gamma }^{(k)} - a_{\boldsymbol{\gamma }}^{(k)}\left\{ H^{-}_{\boldsymbol{\gamma }}P(\boldsymbol{\beta }^{(k+1)}, \boldsymbol{\gamma }^{(k)}, \boldsymbol{\theta }^{(k)})\right\} ^{-1}\nabla _{\boldsymbol{\gamma }}P(\boldsymbol{\beta }^{(k+1)}, \boldsymbol{\gamma }^{(k)}, \boldsymbol{\theta }^{(k)}). \end{aligned}$$

Stepsizes $a_{\boldsymbol{\beta }}^{(k)}$ and $a_{\boldsymbol{\gamma }}^{(k)}$ ensure that each update increases the value of the penalised log-likelihood with respect to $\boldsymbol{\beta }$ and $\boldsymbol{\gamma }$, respectively, holding other parameters fixed.

After updating the estimates of $\boldsymbol{\beta }$ and $\boldsymbol{\gamma }$, the MI algorithm is used to update $\boldsymbol{\theta }$ while constraining $\boldsymbol{\theta }$ to be non-negative as follows:

6 $$\begin{aligned} \boldsymbol{\theta }^{(k+1)} = \boldsymbol{\theta }^{(k)} + a_{\boldsymbol{\theta }}^{(k)}S_{\boldsymbol{\theta }}(\boldsymbol{\beta }^{(k+1)}, \boldsymbol{\gamma }^{(k+1)}, \boldsymbol{\theta }^{(k)}) \nabla _{\boldsymbol{\theta }}P(\boldsymbol{\beta }^{(k+1)}, \boldsymbol{\gamma }^{(k+1)}, \boldsymbol{\theta }^{(k)}), \end{aligned}$$

where $S_{\boldsymbol{\theta }}$ is a diagonal matrix also given in the supplementary material. Similarly, $a_{\boldsymbol{\theta }}^{(k)}$ is the stepsize ensuring an increase in the penalised log-likelihood value with respect to $\boldsymbol{\theta }$ at iteration *k*.

### Automatic smoothing parameter selection

A crucial part of our penalised likelihood approach is the inclusion of an automatic selection process for the smoothing parameter. Traditional methods may require manual tuning of smoothing parameters, which introduces subjectivity into the estimation process. Automatic selection methods help in avoiding this subjectivity by providing an objective and data-driven approach to choose the smoothing parameter. In addition, the choice of smoothing parameter directly impacts the performance of penalised likelihood methods. Selecting an optimal smoothing parameter helps achieve a balance between smoothness of the estimated baseline hazard and overfitting.

We adopt a marginal likelihood-based method to estimate the smoothing parameter *h*. As noted by Cai and Betensky [25], Ma et al. [16] and others, the penalty function can be represented as the log-prior density of $\boldsymbol{\theta }$ where $\boldsymbol{\theta } \sim N (\boldsymbol{0}_{m \times 1}, \sigma _h^2\boldsymbol{R}^{-1})$, with the smoothing parameter introduced through $\sigma _h^2 = 1/{2h}$. Therefore, the penalised log-likelihood is equivalent to the log-posterior density, which is denoted by $\ell _P(\boldsymbol{\beta }, \boldsymbol{\gamma }, \boldsymbol{\theta }, \sigma _h^2)$ henceforth. Applying a Laplace’s method to obtain an approximate marginal likelihood, derived by integrating out $\boldsymbol{\beta }, \boldsymbol{\gamma }$, and $\boldsymbol{\theta }$ from $\exp \{ \ell _P(\boldsymbol{\beta }, \boldsymbol{\gamma }, \boldsymbol{\theta }, \sigma _h^2)\}$, yields an approximate marginal log-likelihood of $\sigma _h^2$:

7 $$\begin{aligned} \ell _m\left( \sigma ^2_h\right) \approx \frac{d}{2}\ln \left( 2\pi \right) -\frac{m}{2}\log \left( \sigma ^2_h\right) + \ell \left( \hat{\boldsymbol{\beta }}, \hat{\boldsymbol{\gamma }}, \hat{\boldsymbol{\theta }}\right) - \frac{1}{2\sigma ^2_h}\hat{\boldsymbol{\theta }}^T\boldsymbol{R}\hat{\boldsymbol{\theta }} - \frac{1}{2}\log |\hat{\boldsymbol{F}} + \boldsymbol{Q}\left( \sigma ^2_h\right) |, \end{aligned}$$

where $\hat{\boldsymbol{F}}$ is the negative Hessian of the log-likelihood $\ell (\boldsymbol{\beta }, \boldsymbol{\gamma }, \boldsymbol{\theta })$ evaluated at $\hat{\boldsymbol{\beta }}, \hat{\boldsymbol{\gamma }}$, and $\hat{\boldsymbol{\theta }}$, and $\boldsymbol{Q}(\sigma ^2_h) = \text {diag}(\textbf{0}_{p\times p}, \textbf{0}_{q\times q}, (1/\sigma ^2_h) \boldsymbol{R})$.

Since $\partial \ell _m(\sigma ^2_h)/\partial \sigma ^2_h$ is non-linear in $\sigma ^2_h$, it requires an optimisation procedure to obtain an accurate estimate of $\sigma ^2_h$. However, the following simple strategy, which yields an approximate $\sigma ^2_h$, works extremely well. Notice that the solution maximising $\ell _m(\sigma ^2_h)$ satisfies

8 $$\begin{aligned} \hat{\sigma }^2_h = \frac{\hat{\boldsymbol{\theta }}^T\boldsymbol{R}\hat{\boldsymbol{\theta }}}{m - \nu }, \end{aligned}$$

where $\nu = \text {tr}\{(\hat{\boldsymbol{F}} + \boldsymbol{Q}(\hat{\sigma }^2_h))^{-1}\boldsymbol{Q}(\hat{\sigma }^2_h)\}$, which can be interpreted as the model degrees of freedom. Hence, we can simply update $\sigma ^2_h$ using this formula where the $\sigma ^2_h$ on the right-hand side is replaced by its most current value.

Since updating the MPL estimates of $\boldsymbol{\beta }, \boldsymbol{\gamma }$, and $\boldsymbol{\theta }$ depends on the estimate of $\sigma ^2_h$, we use an alternating scheme to update all of the parameters. Firstly, with the estimate of $\sigma ^2_h$ fixed, we obtain the estimates of $\boldsymbol{\beta }, \boldsymbol{\gamma }$, and $\boldsymbol{\theta }$ by maximising the penalised log-likelihood. Then, once the values of $\hat{\boldsymbol{\beta }}, \hat{\boldsymbol{\gamma }}$, and $\hat{\boldsymbol{\theta }}$ are obtained, the estimate of $\hat{\sigma }^2_h$ is updated by (8). This process repeats itself until *ν* is stabilised, meaning the fluctuation of *ν* is bounded within a pre-determined tolerance level.

Together, the components in the Computational framework and Automatic smoothing parameter selection sections facilitate convergence despite the added algebraic and computational demands of interval-censored data.

### Asymptotic properties

The asymptotic results in Ma et al. [16] (see also [26], Section 4.4) are sufficiently general to ensure that the MPL estimates of $\boldsymbol{\beta }$, $\boldsymbol{\gamma }$, and $\boldsymbol{\theta }$ are asymptotically consistent, while the asymptotic normality result can be derived under certain regularity conditions with a fixed *m*. For readers interested in the technical connection from previous work to the present setting, a brief outline is provided in Section C of the supplementary material. Throughout this section, *m* is treated as fixed and chosen in advance, so that the asymptotic results are derived in the regime where $n \rightarrow \infty$ with *m* held constant. An extension to a growing-basis regime, where $m \rightarrow \infty$ with *n*, would require a sieve-type asymptotic framework and is left for future work. Accordingly, our focus in this section is on a practically useful large-sample approximate normal distribution for the penalised likelihood estimator of the model parameters, under the regime where *n* is large and *m* is fixed (*m < n*). This approximate distribution depends on both *m* and the smoothing parameter *h*.

Now let $\boldsymbol{\eta }_0 = (\boldsymbol{\theta }_0^T, \boldsymbol{\beta }_0^T, \boldsymbol{\gamma }_0^T)^T$ denote the *(m+p+q) × 1* vector of true parameter values where *m* is fixed. Inference on $\boldsymbol{\beta }$, $\boldsymbol{\gamma }$, and the baseline hazard $\lambda _0(t)$ is based on the asymptotic normality of $\boldsymbol{\eta }$. In practice, some of the $\boldsymbol{\theta } \ge 0$ constraints can be active, which means these $\theta _u = 0$ and their corresponding gradient components are negative. Active constraints often occurs when an excessive number of knots are used. In such instances, the penalty function will suppress unnecessary $\theta _u$ to zero. Ignoring these active constraints can yield issues such as negative asymptotic variances. To address active constraints, we adopt, similar to Ma et al. [16], the constrained asymptotic normality framework of Moore et al. [27]. Specifically, let the first $\tilde{m}$ of the $\boldsymbol{\theta } \ge 0$ constraints be active; then we define

$$\begin{aligned} \boldsymbol{U} = \left[ \begin{array}{ll} \boldsymbol{0}_{(m - \tilde{m} + p + q) \times \tilde{m}}&\boldsymbol{I}_{(m - \tilde{m} + p + q) \times (m - \tilde{m} + p + q)} \end{array}\right] , \end{aligned}$$

which satisfies $\boldsymbol{U}^T\boldsymbol{U} = \boldsymbol{I}$. As stated in Ma [26], assuming that the required assumptions are met, the distribution of $\hat{\boldsymbol{\eta }}$ is approximately multivariate normal when *n* is large. In addition, the covariance matrix can be calculated using

9 $$\begin{aligned} \text {var}(\hat{\boldsymbol{\eta }}) = -\boldsymbol{B}(\hat{\boldsymbol{\eta }})^{-1}\frac{\partial ^2\ell (\hat{\boldsymbol{\eta }})}{\partial \boldsymbol{\eta }\partial \boldsymbol{\eta }^T}\boldsymbol{B}(\hat{\boldsymbol{\eta }})^{-1} \end{aligned}$$

where

$$\begin{aligned} \boldsymbol{B}(\hat{\boldsymbol{\eta }})^{-1} = \boldsymbol{U}\left( \boldsymbol{U}^T\left( -\frac{\partial ^2\ell (\hat{\boldsymbol{\eta }})}{\partial \boldsymbol{\eta }\partial \boldsymbol{\eta }^T} +h\boldsymbol{R}\right) \boldsymbol{U}\right) ^{-1}\boldsymbol{U}^T. \end{aligned}$$

Here, $\boldsymbol{U}$ aids in deleting the rows and columns associated with the active constraints in $-\left( \partial ^2\ell (\hat{\boldsymbol{\eta }})/\partial \boldsymbol{\eta }\partial \boldsymbol{\eta }^T\right) +h\boldsymbol{R}$. Then, $\boldsymbol{B}(\hat{\boldsymbol{\eta }})^{-1}$ is obtained by padding the inverse of $\boldsymbol{U}^T\left\{ -\left( \partial ^2\ell (\hat{\boldsymbol{\eta }})/\partial \boldsymbol{\eta }\partial \boldsymbol{\eta }^T\right) +h\boldsymbol{R}\right\} \boldsymbol{U}$ with zeros in the deleted rows and columns.

In practice, active constraints are identified by examining both the values of $\theta _u$ and their gradients at convergence of the Pseudo Newton-MI algorithm described in this section. Certain $\theta _u$ values may precisely reach zero with negative gradients, clearly designating them as active constraints. However, there may be $\theta _u$ values that are very close to, but not exactly at zero. To account for near-zero estimates, we classify $\theta _u$ as active when $\theta _u < 10^{-3}$ and the corresponding gradient is less than *-ε*, for some threshold *ε> 0*, such as $10^{-2}$. If it happens $\theta _u < 10^{-3}$ but the corresponding gradient is positive after one complete iteration, we then reset $\theta _u=1/2$ and re-run the iterations.

## Simulation

A simulation study was conducted to evaluate the performance of the proposed methodology. To the best of our knowledge, there are currently no alternative approaches or publicly available R packages for fitting semiparametric AFT models with time-varying covariates under partly interval censoring. Accordingly, the simulation focuses solely on assessing the accuracy of the estimates produced by our method. We begin by describing the data generation mechanism.

In this simulation study, two time-fixed covariates were included, with the values of $x_{i1}$ and $x_{i2}$ generated according to a $\text {Bernoulli}(0.5)$ distribution, and a $\text {Unif}[0, 3]$ distribution, respectively. We also included a single time-varying covariate, $z_i(t)$, defined as a discrete function of time containing a single change point: $z_i(t) = 0 \text { if } 0< t < \tau _i, \text { and } z_i(t) = 1 \text { if } t \ge \tau _i$. The change point $\tau _i> 0$, representing the time at which individual *i* received a particular treatment, was drawn according to: $\tau _i \sim \text {Unif}[0,1]$. An alternative approach to generate observations from a time-varying covariate using a continuous function can be found in Section D of the supplementary material.

The baseline hazard was specified by a Weibull hazard given by $\lambda _0(\kappa ) = 3\kappa ^2$, and the corresponding baseline survival function was $S_0(\kappa ) = \exp (-\kappa ^3)$. Therefore, the survival function for an individual *i*, allowing for a potential single change point, could be written as

$$\begin{aligned} S_i(t) = \left\{ \begin{array}{ll} \exp \{-(te^{-\boldsymbol{x}_i\boldsymbol{\beta }})^3\} & \text {if } 0< t < \tau _i;\\ \exp \{-(\tau _ie^{-\boldsymbol{x}_i\boldsymbol{\beta }} +(t- \tau _i)e^{-\boldsymbol{x}_i\boldsymbol{\beta } - \gamma })^3\}& \text {if } t \ge \tau _i. \end{array}\right. \end{aligned}$$

We simulated failure times $t_i$ using an inverse transform sampling approach. For *i = 1… , n*, we first generated a $u_i \sim \text {Unif}[0,1]$. Then, the event time $t_i$ was determined as follows: if $e^{\boldsymbol{x}_i\boldsymbol{\beta }}\root 3 \of {-\log u_i} < \tau _i$ then $t_i = e^{\boldsymbol{x}_i\boldsymbol{\beta }}\root 3 \of {-\log u_i}$; otherwise, $t_i = \tau _i + e^{\gamma }(e^{\boldsymbol{x}_i\boldsymbol{\beta }}\root 3 \of {-\log u_i} - \tau _i)$. After simulating the event time $t_i$, its corresponding interval-censoring times (namely $y_i^L$ and $y_i^R$) were generated according to the following specifications (see, for example, Cai and Betensky [25]):

$$\begin{aligned} y_i^L = t_i^{I\left( U_i^E< \pi ^E\right) }\left( \alpha _LU_i^L\right) ^{I\left( \pi ^E \le U_i^E, \alpha _LU_i^L \le t_i \le \alpha _RU_i^R\right) }\left( \alpha _RU_i^R\right) ^{I\left( \pi ^E \le U_i^E, \alpha _RU_i^R< t_i\right) }0^{I\left( \pi ^E \le U_i^E, t_i <\alpha _LU_i^L\right) } \end{aligned}$$

and

$$\begin{aligned} y_i^R = t_i^{I\left( U_i^E< \pi ^E\right) }\left( \alpha _LU_i^L\right) ^{I\left( \pi ^E \le U_i^E, t_i< \alpha _LU_i^L\right) }\left( \alpha _RU_i^R\right) ^{I\left( \pi ^E \le U_i^E, \alpha _LU_i^L \le t_i \le \alpha _RU_i^R\right) }\infty ^{I\left( \pi ^E \le U_i^E, \alpha _RU_i^R < t_i\right) }, \end{aligned}$$

where $\pi ^E$ denotes the event proportion, and $U_i^E, U_i^L$, and $U_i^R$ are independent standard uniform random variables used to generate the event times and interval censoring times. Additionally, $\alpha _L$ and $\alpha _R$ are scalar parameters that control the width of the censoring intervals, thereby influencing the proportions of events that are left, right or interval-censored.

In this simulation, the true values of $\beta _1$ and $\beta _2$ were set to 1 and -1 respectively, while the true value of *γ* was set to -0.1. Two sample sizes, *n = 100* and *n = 1000*, were chosen to represent both small and large samples. Additionally, we considered two levels of event proportions: $\pi ^E = 0.3$ and $\pi ^E = 0.7$. Let $\pi ^L$, $\pi ^I$, and $\pi ^R$ represent the proportions of left, interval, and right censoring respectively. When $\pi ^E = 0.3$, the average censoring proportions were $\pi ^L = 0.17$, $\pi ^I = 0.20$, and $\pi ^R = 0.33$. Conversely, when $\pi ^E = 0.7$, the averages were $\pi ^L = 0.08$, $\pi ^I = 0.14$, and $\pi ^R = 0.08$.

For the fitted models, the baseline hazard was approximated using Gaussian basis functions as it did not demand boundary values of the $\kappa _i$s’. The number of basis functions used, *m*, was set to the cubic root of the sample size *n*. The knots (in this case, the mean locations of the Gaussian functions) were chosen based on quantiles of the observed times. Parameter estimation was then performed using the Pseudo Newton-MI algorithm described in Maximum penalised likelihood estimation section with boundary values of the $\kappa _i$s’ fixed after 100 iterations.

We present the results of our simulation study in Tables 1 and 2, and compare them with those obtained by fitting an AFT model that only accommodates right-censored observations along with the time-varying covariates, as described in Ma et al. [15]. In that approach, the censoring intervals for left and interval-censored observations were replaced using midpoint imputation and treated these imputed values as event times.

In the tables that follow, we report the bias, Monte Carlo standard deviation (MCSD), average asymptotic standard deviation (AASD) and coverage probabilities (CP) based on *95%* confidence intervals constructed using both MCSDs and AASDs for each approach. Comparing the MCSDs with the corresponding AASDs allows us to assess the accuracy of the asymptotic variance formula given in (9).

**Table 1:** Results obtained for $\hat{\beta }_1$, $\hat{\beta }_2$, and $\hat{\gamma }$ with *n = 100, m = 5* and different event proportions. These results compare the two approaches, one accommodating interval censoring and the other using midpoint imputation, based on 200 repetitions

	Interval-censoring			Midpoint imputation		
	$\pi ^E = 0.3$			$\pi ^E = 0.3$		
	$\hat{\beta }_1$	$\hat{\beta }_2$	$\hat{\gamma }$	$\hat{\beta }_1$	$\hat{\beta }_2$	$\hat{\gamma }$
Bias	−0.0037	−0.0545	−0.1101	−0.4755	0.4455	0.2451
MCSD	0.1088	0.0792	0.2172	0.1115	0.0802	0.2163
AASD	0.1175	0.0612	0.1933	0.0896	0.0486	0.1581
CP (MCSD)	0.950	0.890	0.925	0.005	0	0.785
CP (AASD)	0.965	0.805	0.865	0	0	0.615
	$\pi ^E = 0.7$			$\pi ^E = 0.7$		
	$\hat{\beta }_1$	$\hat{\beta }_2$	$\hat{\gamma }$	$\hat{\beta }_1$	$\hat{\beta }_2$	$\hat{\gamma }$
Bias	−0.0080	−0.0185	−0.0576	−0.3124	0.2881	0.2744
MCSD	0.0904	0.0624	0.1798	0.1286	0.1015	0.1957
AASD	0.0854	0.0450	0.1460	0.0803	0.0452	0.1421
CP (MCSD)	0.955	0.935	0.945	0.280	0.165	0.720
CP (AASD)	0.945	0.840	0.890	0.105	0.055	0.515

**Table 2:** Results obtained for $\hat{\beta }_1$, $\hat{\beta }_2$, and $\hat{\gamma }$ with *n = 1000, m = 10* and different event proportions. These results compare the two approaches, one accommodating interval censoring and the other for midpoint imputation, based on 200 repetitions

	Interval-censoring			Midpoint imputation		
	$\pi ^E = 0.3$			$\pi ^E = 0.3$		
	$\hat{\beta }_1$	$\hat{\beta }_2$	$\hat{\gamma }$	$\hat{\beta }_1$	$\hat{\beta }_2$	$\hat{\gamma }$
Bias	0.0041	−0.0109	−0.0212	−0.4006	0.3858	0.1268
MCSD	0.0346	0.0202	0.0563	0.0346	0.0246	0.0628
AASD	0.0335	0.0195	0.0572	0.0334	0.0186	0.0568
CP (MCSD)	0.940	0.925	0.940	0	0	0.435
CP (AASD)	0.925	0.920	0.955	0	0	0.375
	$\pi ^E = 0.7$			$\pi ^E = 0.7$		
	$\hat{\beta }_1$	$\hat{\beta }_2$	$\hat{\gamma }$	$\hat{\beta }_1$	$\hat{\beta }_2$	$\hat{\gamma }$
Bias	0.0019	0.0030	0.0056	−0.2609	0.2430	0.1837
MCSD	0.0269	0.0182	0.0473	0.0366	0.0260	0.0769
AASD	0.0253	0.0143	0.0448	0.0286	0.0160	0.0499
CP (MCSD)	0.950	0.935	0.960	0	0	0.345
CP (AASD)	0.925	0.890	0.935	0	0	0.095

These results show that the biases are generally small across sample sizes when using the proposed approach that explicitly accounts for interval censoring. In contrast, using the midpoint imputation yields larger biases and lower coverage probabilities, highlighting its limitations. Moreover, for our approach, the coverage probabilities based on the MCSDs are close to the nominal 95%, indicating a proper balance between bias and variability.

We also observe that both the MCSDs and AASDs improve notably with larger sample sizes. These metrics further benefit from higher proportions of exact event times. At *n = 100*, the AASDs closely align with the MCSDs for $\hat{\beta }_1$ and $\hat{\gamma }$, indicating reasonable asymptotic performance even in smaller samples. However, for $\hat{\beta }_2$, some discrepancies between the MCSDs and AASDs remain evident at *n = 100*. These discrepancies diminish with increased sample size, and by *n = 1000*, as shown in Table 2, the AASDs provide an accurate approximation of the Monte Carlo variability across all parameters.

**Fig. 1:**
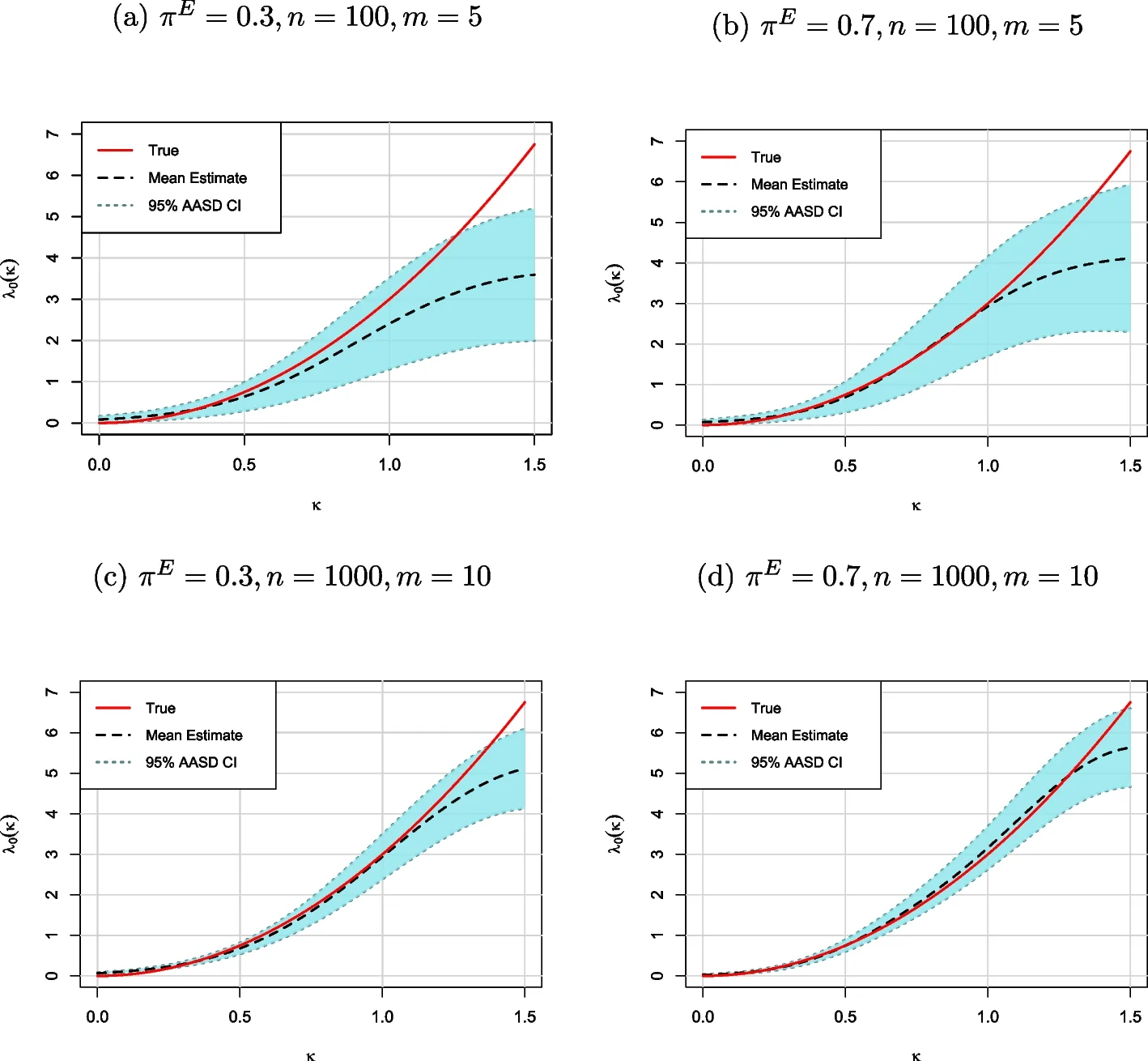
Figures of the baseline hazard functions on a grid of *κ* values. Each plot includes the true baseline hazard curve (solid red line), the mean estimated baseline hazard curve (dashed black line) and area bound by the 95% pointwise confidence interval generated using AASD (coloured region)

**Fig. 2:**
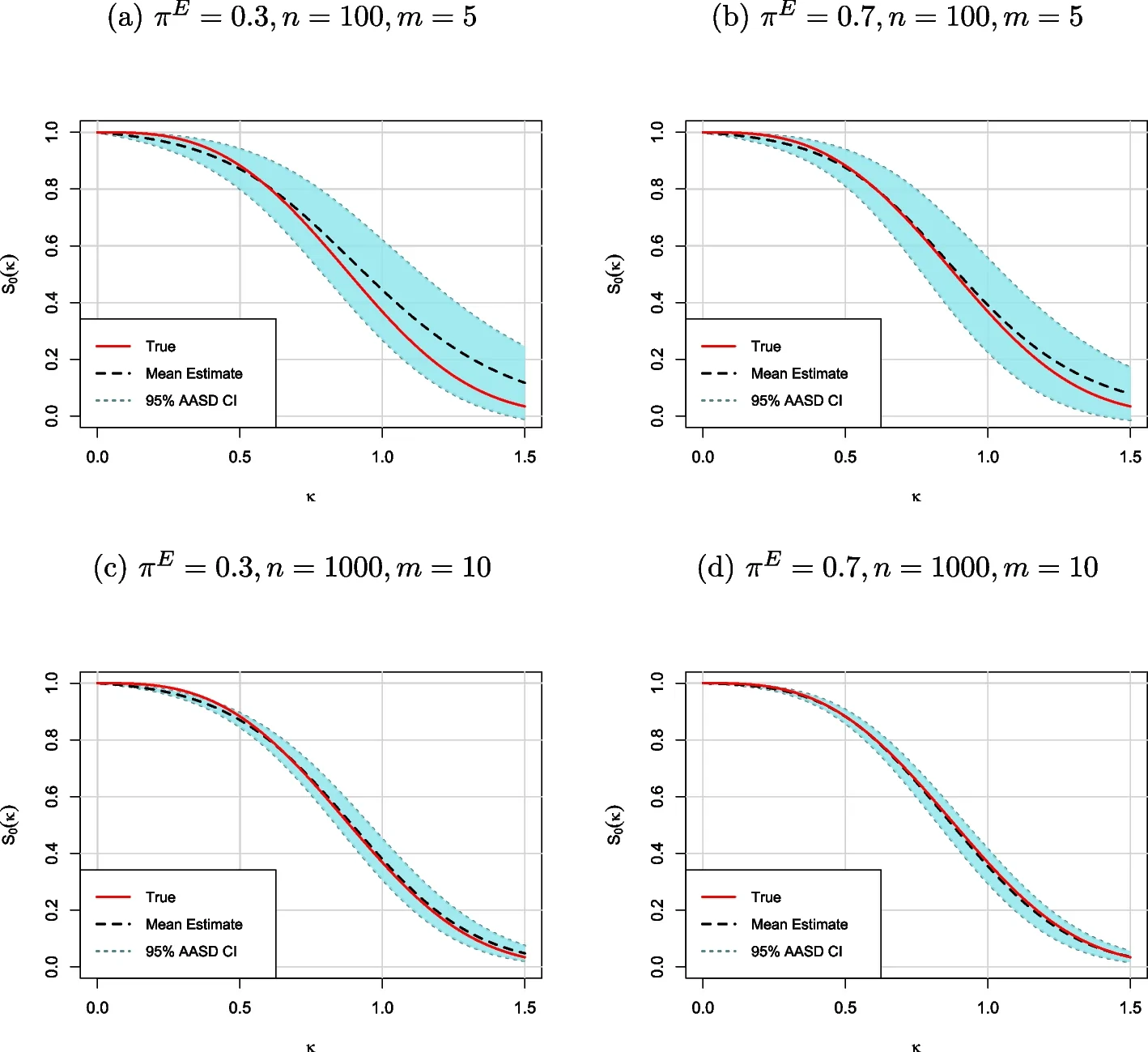
Figures of the baseline survival functions on a grid of *κ* values. Each plot includes the true baseline survival curve (solid red line), the mean estimated baseline survival curve (dashed black line) and area bound by the 95% pointwise confidence interval generated using AASD (coloured region)

Figures 1 and 2 contain plots of the mean estimated baseline hazard and the mean estimated baseline survival, against *κ*, obtained from our approach. The results reveal that the mean estimated baseline hazard and survival functions closely align with their true functions, especially when event proportion is high or the sample size is large. In addition, the widths of the pointwise AASD CIs significantly decrease with an increase in sample size, highlighting improved precision of the estimation. We comment that, in Fig. 1, the deviations between the true and mean estimated baseline hazards toward the ends of the curves are attributed to the fact that, when *n* is small, there are far fewer $\kappa _i$ in this region. This leads to the last Gaussian basis functions having large variance, which causes substantial bias in this region.

The figures in Section E of the supplementary material show extended versions of Figs. 1 and 2, where results from the midpoint imputation approach are also included. The midpoint estimates of the baseline hazard and survival curves are noticeably less accurate across all values of *κ*. The poor performance of the midpoint method is likely due to the relatively wide censoring intervals in the simulated data, which cause inaccurate approximation of event times by the imputed values. These findings further underscore the necessity of our proposed approach, which avoids such approximations and provides more accurate and reliable estimates under interval censoring.

In terms of computational times, each replication in the simulation study with a small sample size (*n = 100*) required about 15 seconds on average to optimise with a MacBook Pro M1 computer. In comparison, the dataset from the WBRTMel trial (*n = 207*) required 3.5 minutes to optimise, while each replication with a large sample size (*n = 1000*) required an average of 5 minutes.

To further assess the robustness of the proposed method, additional simulation results under a log-logistic baseline hazard, illustrating performance under a non-monotone hazard setting, are provided in Section F of the supplementary material.

## Application

### Main analysis

In this section, we re-analyse the WBRTMel trial data, previously studied in Hong et al. [3] by a logistic regression model, using an AFT model adjusted for systemic therapy as a time-varying covariate to assess the effectiveness of WBRT treatment on the time to intracranial failure. The dataset includes basic demographic information and medical records for 207 patients (100 randomly assigned to the WBRT arm and 107 to the observation arm). The time-to-event outcome is the time to intracranial failure (in years). Of the full cohort, 86 patients (41.5%) experienced interval-censored failure times, 28 patients (13.5%) were left-censored, and the remaining 93 (45%) were right-censored. Notably, there were no exactly observed events in this dataset.

The AFT model we use was adjusted for four time-fixed covariates: gender (female or male), presence of extracranial disease at baseline (absent or present), number of melanoma brain metastases (MBM) (1 or 2–3) and age (in years). This AFT model also incorporated a single time-varying covariate representing the status of systemic therapy. During the treatment process, each patient could receive systemic therapy over multiple time periods, with a value of 1 indicating active therapy and 0 indicating inactivity. As such, the covariate values for each patient varied over time, capturing changes in their systemic therapy status. In this dataset, the number of 0’s and 1’s per patient ranged from 1 to 44, with a total of 1,187 recorded values. For 12 patients with missing systemic therapy data, they are more likely to have “inactive” therapy.

**Table 3:** Covariates of interest in the WBRTMel dataset

Covariates	Levels	# Patients	Percentage
Treatment	Observation (reference group)	107	51.7%
	WBRT	100	48.3%
Gender	Female (reference group)	69	33.3%
	Male	138	66.7%
Number of MBM	*=1* (reference group)	127	61.4%
	*>1*	80	38.6%
Extracranial disease	Absent (reference group)	68	32.9%
	Present	139	67.1%
	Value	Mean (median)	Standard deviation
Age	(in years)	61.3 (63.3)	12.2
	Levels	Frequency	
Systemic therapy	0 (Inactive)	1029	
	1 (Active)	158	

A summary of the covariates is provided in Table 3. Approximately two-thirds of the patients were male, with a mean age of 61.3 years (SD=12.2 years). At the time of randomisation, two-thirds of the patients had extracranial disease and nearly 40% had more than one MBM. With regards to the time-varying systemic therapy, in addition to the longitudinal summary of therapy frequency shown in the table, 44% of the patients received systemic therapy at some point during the study.

To fit an AFT model to this data set, the AFT model baseline hazard $\lambda _0(k)$ was approximated using 5 basis functions with the knot locations determined using an empirical quantile function. We note that using more than 5 knots resulted in a higher number of active constraints. The optimisation process was halted once the changes in the estimated regression and basis coefficients were within a tolerance level of $10^{-6}$ and the smoothing parameters had converged.

The results, including the estimated regression coefficients and their corresponding standard errors, are presented in Table 4. Given the asymptotic normality of the estimators for both the regression and basis coefficients, Wald test statistics and associated *p*-values are also reported. From the table, it is evident that the following covariates are statistically significant: treatment group, number of MBM and systemic therapy.

**Table 4:** Results for the covariates in the WBRTMel dataset using an AFT model

	$\hat{\beta }$	se($\hat{\beta }$)	Wald statistic	*P*-value
Treatment (WBRT vs observation)	0.478	0.210	2.279	0.023
Gender (male vs female)	−0.143	0.168	−0.854	0.393
Number of MBM (*> 1* vs *= 1*)	−0.498	0.168	−2.960	0.003
Extracranial disease (present vs absent)	−0.192	0.232	−0.825	0.409
Age (in years)	0.006	0.003	1.644	0.100
	$\hat{\gamma }$	se($\hat{\gamma }$)	Wald statistic	*P*-value
Systemic therapy (active vs inactive)	0.593	0.289	2.050	0.040

**Table 5:** Results for the covariates in the WBRTMel dataset using a Cox model

	$\hat{\beta }$	se($\hat{\beta }$)	Wald statistic	*P*-value
Treatment (WBRT vs observation)	−0.391	0.171	−2.293	0.022
Gender (male vs female)	0.234	0.182	1.287	0.198
Number of MBM (*> 1* vs *= 1*)	0.226	0.171	1.323	0.198
Extracranial disease (present vs absent)	0.090	0.170	0.526	0.599
Age (in years)	−0.003	0.003	−0.918	0.359
	$\hat{\gamma }$	se($\hat{\gamma }$)	Wald statistic	*P*-value
Systemic therapy (active vs inactive)	−0.353	0.001	−398.595	*<2e-16*

For comparison, we also fitted a Cox model for partly-interval censored data with time-varying covariates [18], and the results are presented in Table 5. As expected, the estimated coefficients exhibit opposite signs between the two models. In addition, both models identify the treatment and systemic therapy covariates as statistically significant.

Our findings offer new insights into the efficacy of WBRT in treating advanced melanoma patients. These results, obtained from a semiparametric AFT model adjusted for systemic therapy as a time-varying covariate and other baseline variables such as age, gender, number of MBM and presence of extracranial disease, contrast with the conclusions of Hong et al. [3]. Their study, which utilised a univariate logistic regression model and a Fine and Gray model without covariate adjustment, reported non-effectiveness of WBRT in treating MBM after local therapy.

### Dynamic prediction

Evaluating the efficacy of systemic therapy for individual patients is a key application of the model we developed earlier. One specific form of evaluation can be made through dynamic prediction, which aids clinicians in making informed treatment decisions. In this section, we demonstrate how to calculate the conditional probability that patient *i* survives beyond $\tau _i + dt$, where *dt* reflects the change in time, given that systemic therapy is administered at time $\tau _i$.

For illustrative purposes, we compare the conditional probabilities, as defined in Eq. (11), when individual *i* is given systemic therapy at time $\tau _i \ge 0$ against when they are not given this therapy at $\tau _i$. When given systemic therapy, the therapy is assumed to last for up to three consecutive months, i.e. 3/12 years. Therefore, the simplified form of the time-varying covariate in the WBRTMel dataset, denoted by $z_i(t)$, is given by:

10 $$\begin{aligned} z_i(t) = \left\{ \begin{array}{ll} 0 & \text {if } 0 \le t< \tau _i;\\ 1 & \text {if } \tau _i \le t < \tau _i + 3/12;\\ 0 & \text {if } t \ge \tau _i + 3/12. \end{array}\right. \end{aligned}$$

Note that when systemic therapy is not initiated at time $\tau _i$, then $z_i(t) = 0$ for $t \ge \tau _i$.

In addition, to evaluate the impact of WBRT, we generate conditional probability curves either assuming that the patient received WBRT ($x_{i1} = 1$) or did not receive it ($x_{i1} = 0$). The other covariates are fixed at the following values: Gender = Female ($x_{i2} = 0$), MBM *> 1* ($x_{i3} = 1$), and Extracranial disease = Present ($x_{i4} = 1$). We use four different values of $\tau _i$ for the calculations: $\tau _i = 0$, $\tau _i = 0.5$, $\tau _i = 1$, and $\tau _i = 1.5$.

The conditional probability we wish to calculate is:

11 $$\begin{aligned} P(T_i> \tau _i + dt \mid T_i> \tau _i, \boldsymbol{x}_i, \tilde{\boldsymbol{z}}_i(\tau _i), \tau _i) = \frac{S(\tau _i + dt \mid \boldsymbol{x}_i, \hat{\tilde{\boldsymbol{z}}}_i(\tau _i+dt))}{S(\tau _i \mid \boldsymbol{x}_i, \tilde{\boldsymbol{z}}_i(\tau _i)) }, \end{aligned}$$

with $\boldsymbol{x}_i=(x_{i1}, x_{i2}, x_{i3}, x_{i4})$, and $\tilde{\boldsymbol{z}}_i(t)$ as defined in the Notation section. Here, $\hat{\tilde{\boldsymbol{z}}}_i(t)$ denotes the estimated future trajectory of the time-varying systemic therapy for patient *i*, used for prediction purposes. As this trajectory is pre-specified according to (10), estimation of this quantity is indeed not required.

**Fig. 3:**
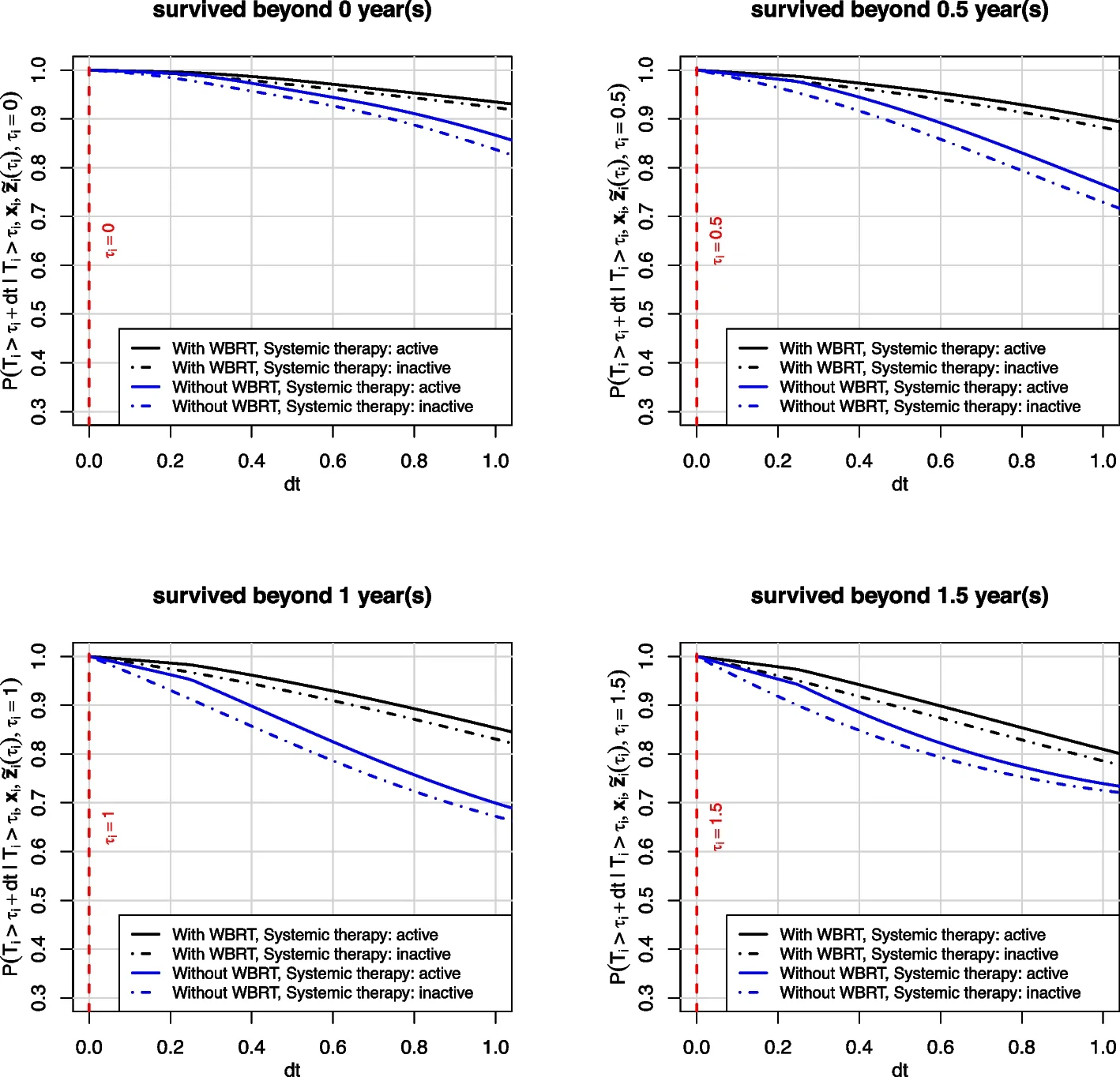
Predicted 1-year effect following initiation of the time-varying systemic therapy (either assumed to be active such that $\boldsymbol{z}_i(\tau _i) = 1$ starting from $\tau _i$ for up to three months, or inactive with $\boldsymbol{z}_i(\tau _i) = 0$). See Eq. (11) on computing brain metastasis-free survival for patients who have survived beyond $\tau _i$ with or without WBRT, for $\tau _i = \{0, 0.5, 1, 1.5\}$; adjusted for other time-fixed covariates: gender as female, number of MBM as *>*1, extracranial disease as present and age as its median value (63.3)

Figure 3 displays the predicted survival probabilities. Across all plots, patients who receive systemic therapy at $\tau _i$ demonstrate higher survival probabilities compared to those who do not. In addition, among patients who receive systemic therapy at $\tau _i$, those who have undergone WBRT show higher predictive survival probabilities than those who have not received WBRT.

Overall, conditional survival probabilities and predictive plots such as those in Fig. 3 can offer clinically meaningful insights, helping to guide decisions regarding the use of systemic treatment.

## Discussion

AFT models facilitate a direct regression analysis of failure times, where each covariate coefficient can be conceived as accelerating or decelerating failure times via a multiplicative factor. In this paper, we focused on the semiparametric AFT models and provided a pathway to estimating such models with time-varying covariates under partly interval censoring, previously unavailable in the literature.

Our approach employs basis functions to approximate the baseline hazard. A roughness penalty was also introduced to enforce smoothness in the baseline hazard estimate. We then proposed an optimisation algorithm that alternates between pseudo-Newton and MI steps to efficiently handle the constrained optimisation problem. Combined with a marginal likelihood-based approach for automatic smoothing parameter selection, the algorithm yields the desired maximum penalised likelihood estimates. The asymptotic results in the Asymptotic properties section provide a theoretical foundation for statistical inference, even in the presence of active constraints. The simulation results demonstrate that the proposed algorithm performs well in terms of estimation accuracy and confidence interval coverage. In contrast, approaches that rely on midpoint imputation for interval-censored data exhibit severe bias and poor coverage.

The R code for the simulation studies and data analysis in this paper, along with a comprehensive vignette for model fitting, is publicly available at https://github.com/aishwarya-b22/AFT_TVC_PIC. In future work, we aim to reduce computational times by incorporating tools such as Rcpp to enhance the efficiency of AFT model implementation.

From a theoretical perspective, the asymptotic results in the Asymptotic properties section are established under a fixed-*m* regime. Extending these results to the case where $m \rightarrow \infty$ with *n*, which would require controlling both the spline approximation error and the estimation error simultaneously, is another direction for future work.

A systematic simulation-based investigation of estimator efficiency under varying censoring compositions and inspection designs, including different interval widths, warrants further study as well.

Exploring alternative formulations based on discrete penalty structures is also of interest, as such approaches may offer improved interpretability and robustness in settings where continuous penalties are less well suited.

Furthermore, in the proposed framework, time-varying covariates are incorporated through the integral term in $\kappa _i(t)$, and inference is conducted conditional on their observed trajectories. This modelling choice avoids the need to specify a joint longitudinal-survival model and leads to a flexible and computationally tractable approach. However, it does not explicitly model the stochastic evolution of the covariates over time.

Extending this framework to jointly model the stochastic evolution of the time-varying covariates alongside the survival process would be a natural direction for future work. In particular, the development of a joint AFT model that simultaneously captures both survival time and longitudinal predictors would be especially relevant in many observational medical studies and clinical trials, where repeated measurements of longitudinal biomarkers often show strong associations with time-to-event outcomes. Such joint models allow for explicit modelling of the dependence between longitudinal processes and survival outcomes, while accounting for potential measurement error in the biomarkers.

Finally, developing robust model selection criteria to effectively compare AFT models with Cox proportional hazards models is an important direction. Given their widespread use in survival analysis and differing assumptions, it is important to establish methods that guide practitioners in selecting the most appropriate model based on model fit and predictive performance. We plan to explore approaches that yield broadly applicable model selection strategies across diverse real-world datasets.

## Supplementary Information

Supplementary Material 1.

## Data Availability

The WBRTMel trial dataset may be accessed upon reasonable request from the Melanoma Institute Australia.

## Abbreviations

AASD: Average Asymptotic Standard Deviation
AFT: Accelerated Failure Time
CI: Confidence Interval
CP: Coverage Probability
MBM: Melanoma Brain Metastases
MCSD: Monte Carlo Standard Deviation
MI: Multiplicative-Iterative
WBRT: Whole Brain Radiotherapy
