# Supplementary material for: Semiparametric accelerated failure time models with time-varying covariates under partly interval censoring
Source: BMC Med Res Methodol. 2026 Jun 13;26:183. doi: 10.1186/s12874-026-02916-y (PMC13487988; doi:10.1186/s12874-026-02916-y)
Supplement: Supplementary file 1 [file 12874_2026_2916_MOESM1_ESM.pdf]

Supplementary Materials for

Semiparametric accelerated failure time models with time-varying covariates under partly interval censoring

by Aishwarya Bhaskaran, Ding Ma, Benoit Liquet, Angela Hong, Stephane Heritier, Serigne N Lo and Jun Ma

A Karush-Kuhn-Tucker conditions

To obtain the constrained solution and compute the penalised maximum likelihood estimates, the necessary Karush-Kuhn-Tucker (KKT) conditions are given by:

$$\begin{aligned}
&\frac{\partial P}{\partial \beta_j} = 0, \quad j = 1 \ldots p;\\
&\frac{\partial P}{\partial \gamma_r} = 0, \quad r = 1 \ldots q;\\
&\frac{\partial P}{\partial \theta_u} = 0 \text{ if } \theta_u > 0 \text{ and } \frac{\partial P}{\partial \theta_u} < 0 \text{ if } \theta_u = 0, \quad u = 1 \ldots m,
\end{aligned}$$

where the case in which $\theta_u = 0$ with a negative gradient signifies an active constraint.

B Vector and matrix representations for the gradients, Hessians and pseudo Hessians

B.1 Notation

Firstly note that by rewriting the survival functions, the explicit form of the log-likelihood, used to evaluate the required gradients and Hessians, can be written as follows

$$\begin{aligned}
\ell(\boldsymbol{\beta}, \boldsymbol{\gamma}, \boldsymbol{\theta}) = \sum_{i=1}^{n} \Big(&\delta_i \{\log \lambda_0(\kappa_i(y_i)) - \boldsymbol{x}_i \boldsymbol{\beta} - \boldsymbol{z}_i(y_i)\boldsymbol{\gamma} - \Lambda_0(\kappa_i(y_i))\} - \delta_i^R \Lambda_0(\kappa_i(y_i^L)) \\
&+ \delta_i^L \log \left[1 - \exp\{-\Lambda_0(\kappa_i(y_i^R))\}\right] \\
&+ \delta_i^I \log \left[\exp\{-\Lambda_0(\kappa_i(y_i^L))\} - \exp\{-\Lambda_0(\kappa_i(y_i^R))\}\right] \Big),
\end{aligned}$$

and recall that the penalised log-likelihood can be written as

$$P(\boldsymbol{\beta}, \boldsymbol{\gamma}, \boldsymbol{\theta}) = \ell(\boldsymbol{\beta}, \boldsymbol{\gamma}, \boldsymbol{\theta}) - h\boldsymbol{\theta}^T \boldsymbol{R} \boldsymbol{\theta}.$$

Now, let us define

$$d_{\boldsymbol{\beta}}P(\boldsymbol{\beta},\boldsymbol{\gamma},\boldsymbol{\theta}), \quad D_{\boldsymbol{\beta}}P(\boldsymbol{\beta},\boldsymbol{\gamma},\boldsymbol{\theta}), \quad \nabla_{\boldsymbol{\beta}}P(\boldsymbol{\beta},\boldsymbol{\gamma},\boldsymbol{\theta}) \quad \text{and} \quad H_{\boldsymbol{\beta}}P(\boldsymbol{\beta},\boldsymbol{\gamma},\boldsymbol{\theta}),$$

as the vector of differentials, derivative vector, gradient and Hessian of the penalized log-likelihood, $P(\boldsymbol{\beta},\boldsymbol{\gamma},\boldsymbol{\theta})$, with respect to $\boldsymbol{\beta}$. In addition, let

$$H^{-}_{\boldsymbol{\beta}}P(\boldsymbol{\beta},\boldsymbol{\gamma},\boldsymbol{\theta}),$$

denote the pseudo Hessian of the penalized log-likelihood with respect to $\boldsymbol{\beta}$. The pseudo Hessian is obtained from the original expression of the Hessian by only extracting the negative terms.

The notation in this subsection applies to other model parameters as well.

B.2 Expressions with respect to $\boldsymbol{\beta}$

Firstly note that,

$$\begin{aligned} d_{\boldsymbol{\beta}}\kappa_i(y_i) &= e^{-\boldsymbol{X}_i\boldsymbol{\beta}}\int_0^{y_i} e^{-z_i(s)\boldsymbol{\gamma}}ds\; d(-\boldsymbol{X}_i\boldsymbol{\beta}) \\ &= -\kappa_i(y_i)\boldsymbol{X}_i d\boldsymbol{\beta}, \end{aligned}$$

and

$$\begin{aligned} d_{\boldsymbol{\beta}}\exp\{-\Lambda_0(\kappa_i(y_i))\} &= -\exp\{-\Lambda_0(\kappa_i(y_i))\}d\{\Lambda_0(\kappa_i(y_i))\} \\ &= -\exp\{-\Lambda_0(\kappa_i(y_i))\}\lambda_0(\kappa_i(y_i))d\{\kappa_i(y_i)\} \\ &= \exp\{-\Lambda_0(\kappa_i(y_i))\}\lambda_0(\kappa_i(y_i))\kappa_i(y_i)\boldsymbol{X}_i d\boldsymbol{\beta}. \end{aligned}$$

B.2.1 Gradient with respect to $\boldsymbol{\beta}$

The gradient of $P(\boldsymbol{\beta},\boldsymbol{\gamma},\boldsymbol{\theta})$ with respect to $\boldsymbol{\beta}$ can be derived as,

$$\nabla_{\boldsymbol{\beta}}P(\boldsymbol{\beta},\boldsymbol{\gamma},\boldsymbol{\theta}) = D_{\boldsymbol{\beta}}P(\boldsymbol{\beta},\boldsymbol{\gamma},\boldsymbol{\theta})^T,$$

which can be shown to be,

$$\begin{aligned} &D_{\boldsymbol{\beta}}P(\boldsymbol{\beta},\boldsymbol{\gamma},\boldsymbol{\theta})^T \\ &= \sum_{i=1}^{n}\boldsymbol{X}_i^T\Bigg(\delta_i\left\{-\frac{\lambda_0'(\kappa_i(y_i))\kappa_i(y_i)}{\lambda_0(\kappa_i(y_i))} - 1 + \lambda_0(\kappa_i(y_i))\kappa_i(y_i)\right\} + \delta_i^R\lambda_0(\kappa_i(y_i^L))\kappa_i(y_i^L)\Bigg) \\ &\quad - \delta_i^L\left[\frac{\exp\{-\Lambda_0(\kappa_i(y_i^R))\}\lambda_0(\kappa_i(y_i^R))\kappa_i(y_i^R)}{1-\exp\{-\Lambda_0(\kappa_i(y_i^R))\}}\right] \\ &\quad + \delta_i^I\left[\frac{\exp\{-\Lambda_0(\kappa_i(y_i^L))\}\lambda_0(\kappa_i(y_i^L))\kappa_i(y_i^L) - \exp\{-\Lambda_0(\kappa_i(y_i^R))\}\lambda_0(\kappa_i(y_i^R))\kappa_i(y_i^R)}{\exp\{-\Lambda_0(\kappa_i(y_i^L))\} - \exp\{-\Lambda_0(\kappa_i(y_i^R))\}}\right]\Bigg). \end{aligned}$$

B.2.2 Hessian with respect to $\boldsymbol{\beta}$

Note that

$$H_{\boldsymbol{\beta}}P(\boldsymbol{\beta},\boldsymbol{\gamma},\boldsymbol{\theta}) = D_{\boldsymbol{\beta}}\left(D_{\boldsymbol{\beta}}P(\boldsymbol{\beta},\boldsymbol{\gamma},\boldsymbol{\theta})^T\right).$$

Then the Hessian of $P(\boldsymbol{\beta},\boldsymbol{\gamma},\boldsymbol{\theta})$ with respect to $\boldsymbol{\beta}$ can be shown to be,

$$\begin{aligned}
&H_{\boldsymbol{\beta}}P(\boldsymbol{\beta},\boldsymbol{\gamma},\boldsymbol{\theta})\\
&=\sum_{i=1}^{n}\boldsymbol{X}_i^T\Bigg[\delta_i\Bigg(\left[\frac{\lambda_0(\kappa_i(y_i))\lambda_0^{''}(\kappa_i(y_i))-\{\lambda_0^{'}(\kappa_i(y_i))\}^2}{\{\lambda_0(\kappa_i(y_i))\}^2}-\lambda_0^{'}(\kappa_i(y_i))\right]\{\kappa_i(y_i)\}^2+\left\{\frac{\lambda_0^{'}(\kappa_i(y_i))}{\lambda_0(\kappa_i(y_i))}-\lambda_0(\kappa_i(y_i))\right\}\kappa_i(y_i)\Bigg)\\
&\quad-\delta_i^R\{\lambda_0^{'}(\kappa_i(y_i^L))\kappa_i(y_i^L)+\lambda_0(\kappa_i(y_i^L))\}\kappa_i(y_i^L)-\delta_i^L\Bigg\{\Bigg(\frac{\exp\{-\Lambda_0(\kappa_i(y_i^R))\}\left[\{\lambda_0(\kappa_i(y_i^R))\}^2-\lambda_0^{'}(\kappa_i(y_i^R))\right]}{1-\exp\{-\Lambda_0(\kappa_i(y_i^R))\}}\\
&\quad+\left[\frac{\exp\{-\Lambda_0(\kappa_i(y_i^R))\}\lambda_0(\kappa_i(y_i^R))}{1-\exp\{-\Lambda_0(\kappa_i(y_i^R))\}}\right]^2\Bigg)\{\kappa_i(y_i^R)\}^2-\left[\frac{\exp\{-\Lambda_0(\kappa_i(y_i^R))\}\lambda_0(\kappa_i(y_i^R))}{1-\exp\{-\Lambda_0(\kappa_i(y_i^R))\}}\right]\kappa_i(y_i^R)\Bigg\}\\
&\quad+\delta_i^I\Bigg\{\Bigg(\frac{\exp\{-\Lambda_0(\kappa_i(y_i^L))\}\left[\{\lambda_0(\kappa_i(y_i^L))\}^2-\lambda_0^{'}(\kappa_i(y_i^L))\right]}{\exp\{-\Lambda_0(\kappa_i(y_i^L))\}-\exp\{-\Lambda_0(\kappa_i(y_i^R))\}}-\left[\frac{\exp\{-\Lambda_0(\kappa_i(y_i^L))\}\lambda_0(\kappa_i(y_i^L))}{\exp\{-\Lambda_0(\kappa_i(y_i^L))\}-\exp\{-\Lambda_0(\kappa_i(y_i^R))\}}\right]^2\Bigg)\{\kappa_i(y_i^L)\}^2\\
&\quad-\left[\frac{\exp\{-\Lambda_0(\kappa_i(y_i^L))\}\lambda_0(\kappa_i(y_i^L))}{\exp\{-\Lambda_0(\kappa_i(y_i^L))\}-\exp\{-\Lambda_0(\kappa_i(y_i^R))\}}\right]\kappa_i(y_i^L)-\Bigg(\frac{\exp\{-\Lambda_0(\kappa_i(y_i^R))\}\left[\{\lambda_0(\kappa_i(y_i^R))\}^2-\lambda_0^{'}(\kappa_i(y_i^R))\right]}{\exp\{-\Lambda_0(\kappa_i(y_i^L))\}-\exp\{-\Lambda_0(\kappa_i(y_i^R))\}}\\
&\quad+\left[\frac{\exp\{-\Lambda_0(\kappa_i(y_i^R))\}\lambda_0(\kappa_i(y_i^R))}{\exp\{-\Lambda_0(\kappa_i(y_i^L))\}-\exp\{-\Lambda_0(\kappa_i(y_i^R))\}}\right]^2\Bigg)\{\kappa_i(y_i^R)\}^2+\left[\frac{\exp\{-\Lambda_0(\kappa_i(y_i^R))\}\lambda_0(\kappa_i(y_i^R))}{\exp\{-\Lambda_0(\kappa_i(y_i^L))\}-\exp\{-\Lambda_0(\kappa_i(y_i^R))\}}\right]\kappa_i(y_i^R)\\
&\quad+2\left(\frac{\exp\{-\Lambda_0(\kappa_i(y_i^L))\}\lambda_0(\kappa_i(y_i^L))\kappa_i(y_i^L)\exp\{-\Lambda_0(\kappa_i(y_i^R))\}\lambda_0(\kappa_i(y_i^R))\kappa_i(y_i^R)}{\left[\exp\{-\Lambda_0(\kappa_i(y_i^L))\}-\exp\{-\Lambda_0(\kappa_i(y_i^R))\}\right]^2}\right)\Bigg\}\Bigg]\boldsymbol{X}_i.
\end{aligned}$$

B.2.3 Pseudo Hessian with respect to $\boldsymbol{\beta}$

Subsequently, the pseudo Hessian of $P(\boldsymbol{\beta},\boldsymbol{\gamma},\boldsymbol{\theta})$ with respect to $\boldsymbol{\beta}$ is,

$$\begin{aligned}
&H_{\boldsymbol{\beta}}^{-}P(\boldsymbol{\beta},\boldsymbol{\gamma},\boldsymbol{\theta})\\
&=-\sum_{i=1}^{n}\boldsymbol{X}_i^T\Bigg[\delta_i\left[\left\{\frac{\lambda_0^{'}(\kappa_i(y_i))\kappa_i(y_i)}{\lambda_0(\kappa_i(y_i))}\right\}^2+\lambda_0(\kappa_i(y_i))\kappa_i(y_i)\right]+\delta_i^R\lambda_0(\kappa_i(y_i^L))\kappa_i(y_i^L)\\
&\quad+\delta_i^L\Bigg\{\Bigg(\frac{\exp\{-\Lambda_0(\kappa_i(y_i^R))\}\{\lambda_0(\kappa_i(y_i^R))\}^2}{1-\exp\{-\Lambda_0(\kappa_i(y_i^R))\}}+\left[\frac{\exp\{-\Lambda_0(\kappa_i(y_i^R))\}\lambda_0(\kappa_i(y_i^R))}{1-\exp\{-\Lambda_0(\kappa_i(y_i^R))\}}\right]^2\Bigg)\{\kappa_i(y_i^R)\}^2\Bigg\}\\
&\quad+\delta_i^I\Bigg\{\left[\frac{\exp\{-\Lambda_0(\kappa_i(y_i^L))\}\lambda_0(\kappa_i(y_i^L))}{\exp\{-\Lambda_0(\kappa_i(y_i^L))\}-\exp\{-\Lambda_0(\kappa_i(y_i^R))\}}\right]^2\{\kappa_i(y_i^L)\}^2+\left[\frac{\exp\{-\Lambda_0(\kappa_i(y_i^L))\}\lambda_0(\kappa_i(y_i^L))}{\exp\{-\Lambda_0(\kappa_i(y_i^L))\}-\exp\{-\Lambda_0(\kappa_i(y_i^R))\}}\right]\kappa_i(y_i^L)\\
&\quad+\Bigg(\frac{\exp\{-\Lambda_0(\kappa_i(y_i^R))\}\{\lambda_0(\kappa_i(y_i^R))\}^2}{\exp\{-\Lambda_0(\kappa_i(y_i^L))\}-\exp\{-\Lambda_0(\kappa_i(y_i^R))\}}+\left[\frac{\exp\{-\Lambda_0(\kappa_i(y_i^R))\}\lambda_0(\kappa_i(y_i^R))}{\exp\{-\Lambda_0(\kappa_i(y_i^L))\}-\exp\{-\Lambda_0(\kappa_i(y_i^R))\}}\right]^2\Bigg)\{\kappa_i(y_i^R)\}^2\Bigg]\boldsymbol{X}_i.
\end{aligned}$$

B.3 Expressions with respect to $\boldsymbol{\gamma}$

Next note that,

$$\begin{aligned}
d_{\boldsymbol{\gamma}}\kappa_i(y_i) &= e^{-\boldsymbol{X}_i\boldsymbol{\beta}}\int_0^{y_i}e^{-z_i(s)\boldsymbol{\gamma}}d(-z_i(s)\boldsymbol{\gamma})ds\\
&= -e^{-\boldsymbol{X}_i\boldsymbol{\beta}}\int_0^{y_i}e^{-z_i(s)\boldsymbol{\gamma}}z_i(s)dsd\boldsymbol{\gamma},\\
D_{\boldsymbol{\gamma}}\kappa_i(y_i) &= -e^{-\boldsymbol{X}_i\boldsymbol{\beta}}\int_0^{y_i}e^{-z_i(s)\boldsymbol{\gamma}}z_i(s)ds,
\end{aligned}$$

and

$$D_{\boldsymbol{\gamma}}\{D_{\boldsymbol{\gamma}}\kappa_i(y_i)^T\} = e^{-\boldsymbol{X}_i\boldsymbol{\beta}}\int_0^{y_i} e^{-z_i(s)\boldsymbol{\gamma}} z_i(s)^T z_i(s) ds.$$

Also note that,

$$d\exp\{-\Lambda_0(\kappa_i(y_i))\} = -\exp\{-\Lambda_0(\kappa_i(y_i))\}\lambda_0(\kappa_i(y_i))d_{\boldsymbol{\gamma}}\kappa_i(y_i).$$

B.3.1 Gradient with respect to γ

The gradient of $P(\boldsymbol{\beta}, \boldsymbol{\gamma}, \boldsymbol{\theta})$ with respect to $\boldsymbol{\gamma}$ can be derived as,

$$\nabla_{\boldsymbol{\gamma}} P(\boldsymbol{\beta}, \boldsymbol{\gamma}, \boldsymbol{\theta}) = D_{\boldsymbol{\gamma}} P(\boldsymbol{\beta}, \boldsymbol{\gamma}, \boldsymbol{\theta})^T$$

which can be shown to be,

$$\begin{aligned}
&D_{\boldsymbol{\gamma}} P(\boldsymbol{\beta}, \boldsymbol{\gamma}, \boldsymbol{\theta})^T \\
&= \sum_{i=1}^{n} \Bigg(- \delta_i z_i(y_i)^T + \delta_i \left\{ \frac{\lambda_0'(\kappa_i(y_i))}{\lambda_0(\kappa_i(y_i))} - \lambda_0(\kappa_i(y_i)) \right\} D_{\boldsymbol{\gamma}}\kappa_i(y_i)^T - \delta_i^R \lambda_0(\kappa_i(y_i^L)) D_{\boldsymbol{\gamma}}\kappa_i(y_i^L)^T \\
&\quad + \delta_i^L \left[\frac{\exp\{-\Lambda_0(\kappa_i(y_i^R))\}\lambda_0(\kappa_i(y_i^R))}{1 - \exp\{-\Lambda_0(\kappa_i(y_i^R))\}} \right] D_{\boldsymbol{\gamma}}\kappa_i(y_i^R)^T \\
&\quad + \delta_i^I \left[\frac{-\exp\{-\Lambda_0(\kappa_i(y_i^L))\}\lambda_0(\kappa_i(y_i^L)) D_{\boldsymbol{\gamma}}\kappa_i(y_i^L)^T + \exp\{-\Lambda_0(\kappa_i(y_i^R))\}\lambda_0(\kappa_i(y_i^R)) D_{\boldsymbol{\gamma}}\kappa_i(y_i^R)^T}{\exp\{-\Lambda_0(\kappa_i(y_i^L)) - \exp\{-\Lambda_0(\kappa_i(y_i^R))\}} \right] \Bigg).
\end{aligned}$$

B.3.2 Hessian with respect to γ

Similar to the computation of $H_{\boldsymbol{\beta}} P(\boldsymbol{\beta}, \boldsymbol{\gamma}, \boldsymbol{\theta})$, note that

$$H_{\boldsymbol{\gamma}} P(\boldsymbol{\beta}, \boldsymbol{\gamma}, \boldsymbol{\theta}) = D_{\boldsymbol{\gamma}} \left(D_{\boldsymbol{\gamma}} P(\boldsymbol{\beta}, \boldsymbol{\gamma}, \boldsymbol{\theta})^T \right).$$

Then the Hessian of $P(\boldsymbol{\beta}, \boldsymbol{\gamma}, \boldsymbol{\theta})$ with respect to $\boldsymbol{\gamma}$ can be shown to be,

$$
\begin{aligned}
&H_{\boldsymbol{\gamma}} P(\boldsymbol{\beta}, \boldsymbol{\gamma}, \boldsymbol{\theta}) \\
&= \sum_{i=1}^{n} \Bigg[\delta_i \Bigg(D_{\boldsymbol{\gamma}} \kappa_i(y_i)^T \left[\frac{\lambda_0(\kappa_i(y_i)) \lambda_0^{''}(\kappa_i(y_i)) - \{\lambda_0^{'}(\kappa_i(y_i))\}^2}{\{\lambda_0(\kappa_i(y_i))\}^2} - \lambda_0^{'}(\kappa_i(y_i)) \right] D_{\boldsymbol{\gamma}} \kappa_i(y_i) \\
&\quad + \left\{ \frac{\lambda_0^{'}(\kappa_i(y_i))}{\lambda_0(\kappa_i(y_i))} - \lambda_0(\kappa_i(y_i)) \right\} D\{D_{\boldsymbol{\gamma}} \kappa_i(y_i)^T\} \Bigg) - \delta_i^R \left[\lambda_0(\kappa_i(y_i^L)) D\{D_{\boldsymbol{\gamma}} \kappa_i(y_i^L)^T\} + D_{\boldsymbol{\gamma}} \kappa_i(y_i^L)^T \lambda_0^{'}(\kappa_i(y_i^L)) D_{\boldsymbol{\gamma}} \kappa_i(y_i^L) \right] \\
&\quad + \delta_i^L \Bigg\{ \left[\frac{\exp\{-\Lambda_0(\kappa_i(y_i^R))\} \lambda_0(\kappa_i(y_i^R))}{1 - \exp\{-\Lambda_0(\kappa_i(y_i^R))\}} \right] D\{D_{\boldsymbol{\gamma}} \kappa_i(y_i^R)^T\} \\
&\quad + D_{\boldsymbol{\gamma}} \kappa_i(y_i^R)^T \left(\frac{\exp\{-\Lambda_0(\kappa_i(y_i^R))\} \left[\lambda_0^{'}(\kappa_i(y_i^R)) - \{\lambda_0(\kappa_i(y_i^R))\}^2 \right]}{1 - \exp\{-\Lambda_0(\kappa_i(y_i^R))\}} - \left[\frac{\exp\{-\Lambda_0(\kappa_i(y_i^R))\} \lambda_0(\kappa_i(y_i^R))}{1 - \exp\{-\Lambda_0(\kappa_i(y_i^R))\}} \right]^2 \right) D_{\boldsymbol{\gamma}} \kappa_i(y_i^R) \Bigg\} \\
&\quad + \delta_i^I \Bigg\{ D_{\boldsymbol{\gamma}} \kappa_i(y_i^L)^T \left(\frac{\exp\{-\Lambda_0(\kappa_i(y_i^L))\} \left[\{\lambda_0(\kappa_i(y_i^L))\}^2 - \lambda_0^{'}(\kappa_i(y_i^L)) \right]}{\exp\{-\Lambda_0(\kappa_i(y_i^L))\} - \exp\{-\Lambda_0(\kappa_i(y_i^R))\}} - \left[\frac{\exp\{-\Lambda_0(\kappa_i(y_i^L))\} \lambda_0(\kappa_i(y_i^L))}{\exp\{-\Lambda_0(\kappa_i(y_i^L))\} - \exp\{-\Lambda_0(\kappa_i(y_i^R))\}} \right]^2 \right) \\
&\quad \times D_{\boldsymbol{\gamma}} \kappa_i(y_i^L) - \left[\frac{\exp\{-\Lambda_0(\kappa_i(y_i^L))\} \lambda_0(\kappa_i(y_i^L))}{\exp\{-\Lambda_0(\kappa_i(y_i^L))\} - \exp\{-\Lambda_0(\kappa_i(y_i^R))\}} \right] D\{D_{\boldsymbol{\gamma}} \kappa_i(y_i^L)^T\} \\
&\quad - D_{\boldsymbol{\gamma}} \kappa_i(y_i^R)^T \left(\frac{\exp\{-\Lambda_0(\kappa_i(y_i^R))\} \left[\{\lambda_0(\kappa_i(y_i^R))\}^2 - \lambda_0^{'}(\kappa_i(y_i^R)) \right]}{\exp\{-\Lambda_0(\kappa_i(y_i^L))\} - \exp\{-\Lambda_0(\kappa_i(y_i^R))\}} + \left[\frac{\exp\{-\Lambda_0(\kappa_i(y_i^R))\} \lambda_0(\kappa_i(y_i^R))}{\exp\{-\Lambda_0(\kappa_i(y_i^L))\} - \exp\{-\Lambda_0(\kappa_i(y_i^R))\}} \right]^2 \right) \\
&\quad \times D_{\boldsymbol{\gamma}} \kappa_i(y_i^R) + \left[\frac{\exp\{-\Lambda_0(\kappa_i(y_i^R))\} \lambda_0(\kappa_i(y_i^R))}{\exp\{-\Lambda_0(\kappa_i(y_i^L))\} - \exp\{-\Lambda_0(\kappa_i(y_i^R))\}} \right] D\{D_{\boldsymbol{\gamma}} \kappa_i(y_i^R)^T\} \\
&\quad + D_{\boldsymbol{\gamma}} \kappa_i(y_i^L)^T \left(\frac{\exp\{-\Lambda_0(\kappa_i(y_i^L))\} \lambda_0(\kappa_i(y_i^L)) \exp\{-\Lambda_0(\kappa_i(y_i^R))\} \lambda_0(\kappa_i(y_i^R))}{\left[\exp\{-\Lambda_0(\kappa_i(y_i^L))\} - \exp\{-\Lambda_0(\kappa_i(y_i^R))\}\right]^2} \right) D_{\boldsymbol{\gamma}} \kappa_i(y_i^R) \\
&\quad + D_{\boldsymbol{\gamma}} \kappa_i(y_i^R)^T \left(\frac{\exp\{-\Lambda_0(\kappa_i(y_i^R))\} \lambda_0(\kappa_i(y_i^R)) \exp\{-\Lambda_0(\kappa_i(y_i^L))\} \lambda_0(\kappa_i(y_i^L))}{\left[\exp\{-\Lambda_0(\kappa_i(y_i^L))\} - \exp\{-\Lambda_0(\kappa_i(y_i^R))\}\right]^2} \right) D_{\boldsymbol{\gamma}} \kappa_i(y_i^L) \Bigg\} \Bigg].
\end{aligned}
$$

B.3.3 Pseudo Hessian with respect to $\boldsymbol{\gamma}$

Hence, the pseudo Hessian of $P(\boldsymbol{\beta}, \boldsymbol{\gamma}, \boldsymbol{\theta})$ with respect to $\boldsymbol{\gamma}$ can be derived as,

$$
\begin{aligned}
&H_{\boldsymbol{\gamma}}^{-} P(\boldsymbol{\beta}, \boldsymbol{\gamma}, \boldsymbol{\theta}) \\
&= -\sum_{i=1}^{n} \Bigg[\delta_i \left(D_{\boldsymbol{\gamma}} \kappa_i(y_i)^T \left\{ \frac{\lambda_0^{'}(\kappa_i(y_i))}{\lambda_0(\kappa_i(y_i))} \right\}^2 D_{\boldsymbol{\gamma}} \kappa_i(y_i) + \lambda_0(\kappa_i(y_i)) D\{D_{\boldsymbol{\gamma}} \kappa_i(y_i)^T\} \right) - \delta_i^R \lambda_0(\kappa_i(y_i^L)) D\{D_{\boldsymbol{\gamma}} \kappa_i(y_i^L)^T\} \\
&\quad + \delta_i^L \left\{ D_{\boldsymbol{\gamma}} \kappa_i(y_i^R)^T \left(\frac{\exp\{-\Lambda_0(\kappa_i(y_i^R))\} \{\lambda_0(\kappa_i(y_i^R))\}^2}{1 - \exp\{-\Lambda_0(\kappa_i(y_i^R))\}} + \left[\frac{\exp\{-\Lambda_0(\kappa_i(y_i^R))\} \lambda_0(\kappa_i(y_i^R))}{1 - \exp\{-\Lambda_0(\kappa_i(y_i^R))\}} \right]^2 \right) D_{\boldsymbol{\gamma}} \kappa_i(y_i^R) \right\} \\
&\quad + \delta_i^I \Bigg\{ D_{\boldsymbol{\gamma}} \kappa_i(y_i^L)^T \left[\frac{\exp\{-\Lambda_0(\kappa_i(y_i^L))\} \lambda_0(\kappa_i(y_i^L))}{\exp\{-\Lambda_0(\kappa_i(y_i^L))\} - \exp\{-\Lambda_0(\kappa_i(y_i^R))\}} \right]^2 D_{\boldsymbol{\gamma}} \kappa_i(y_i^L) \\
&\quad + \left[\frac{\exp\{-\Lambda_0(\kappa_i(y_i^L))\} \lambda_0(\kappa_i(y_i^L))}{\exp\{-\Lambda_0(\kappa_i(y_i^L))\} - \exp\{-\Lambda_0(\kappa_i(y_i^R))\}} \right] D\{D_{\boldsymbol{\gamma}} \kappa_i(y_i^L)^T\} \\
&\quad + D_{\boldsymbol{\gamma}} \kappa_i(y_i^R)^T \left(\frac{\exp\{-\Lambda_0(\kappa_i(y_i^R))\} \{\lambda_0(\kappa_i(y_i^R))\}^2}{\exp\{-\Lambda_0(\kappa_i(y_i^L))\} - \exp\{-\Lambda_0(\kappa_i(y_i^R))\}} + \left[\frac{\exp\{-\Lambda_0(\kappa_i(y_i^R))\} \lambda_0(\kappa_i(y_i^R))}{\exp\{-\Lambda_0(\kappa_i(y_i^L))\} - \exp\{-\Lambda_0(\kappa_i(y_i^R))\}} \right]^2 \right) D_{\boldsymbol{\gamma}} \kappa_i(y_i^R) \Bigg\} \Bigg].
\end{aligned}
$$

B.4 Expressions with respect to $\boldsymbol{\theta}$

Let

$$\boldsymbol{\psi}(\kappa_i(y_i)) = [\psi_1(\kappa_i(y_i)), \ldots, \psi_m(\kappa_i(y_i))]^T \quad \text{and} \quad \boldsymbol{\Psi}(\kappa_i(y_i)) = [\Psi_1(\kappa_i(y_i)), \ldots, \Psi_m(\kappa_i(y_i))]^T.$$

Also note that,

$$d\lambda_0(\kappa_i(y_i)) = \boldsymbol{\psi}(\kappa_i(y_i))d\boldsymbol{\theta} \quad \text{and} \quad d\Lambda_0(\kappa_i(y_i)) = \boldsymbol{\Psi}(\kappa_i(y_i))d\boldsymbol{\theta}.$$

B.4.1 Gradient with respect to $\boldsymbol{\theta}$

The gradient of $P(\boldsymbol{\beta}, \boldsymbol{\gamma}, \boldsymbol{\theta})$ with respect to $\boldsymbol{\gamma}$ can be derived as,

$$\nabla_{\boldsymbol{\theta}} P(\boldsymbol{\beta}, \boldsymbol{\gamma}, \boldsymbol{\theta}) = D_{\boldsymbol{\theta}} P(\boldsymbol{\beta}, \boldsymbol{\gamma}, \boldsymbol{\theta})^T$$

which can be shown to be,

$$\begin{aligned}
&D_{\boldsymbol{\theta}} P(\boldsymbol{\beta}, \boldsymbol{\gamma}, \boldsymbol{\theta})^T \\
&= \sum_{i=1}^{n} \Bigg(\delta_i \left\{ \frac{1}{\lambda_0(\kappa_i(y_i))} \boldsymbol{\psi}(\kappa_i(y_i)) - \boldsymbol{\Psi}(\kappa_i(y_i)) \right\} - \delta_i^R \boldsymbol{\Psi}(\kappa_i(y_i^L)) + \delta_i^L \left[\frac{\exp\{-\Lambda_0(\kappa_i(y_i^R))\}}{1 - \exp\{-\Lambda_0(\kappa_i(y_i^R))\}} \right] \boldsymbol{\Psi}(\kappa_i(y_i)) \\
&\quad + \delta_i^I \left[\frac{1}{\exp\{-\Lambda_0(\kappa_i(y_i^L))\} - \exp\{-\Lambda_0(\kappa_i(y_i^R))\}} \right] \Big[- \boldsymbol{\Psi}(\kappa_i(y_i^L)) \exp\{-\Lambda_0(\kappa_i(y_i^L))\} \\
&\quad + \boldsymbol{\Psi}(\kappa_i(y_i^R)) \exp\{-\Lambda_0(\kappa_i(y_i^R))\} \Big] \Bigg) - 2h\boldsymbol{\theta}^T \boldsymbol{R}.
\end{aligned}$$

B.4.2 Hessian with respect to $\boldsymbol{\theta}$

We have that,

$$H_{\boldsymbol{\theta}} P(\boldsymbol{\beta}, \boldsymbol{\gamma}, \boldsymbol{\theta}) = D_{\boldsymbol{\theta}} \left(D_{\boldsymbol{\theta}} P(\boldsymbol{\beta}, \boldsymbol{\gamma}, \boldsymbol{\theta})^T \right).$$

Then the Hessian of $P(\boldsymbol{\beta}, \boldsymbol{\gamma}, \boldsymbol{\theta})$ with respect to $\boldsymbol{\theta}$ can be derived as,

$$\begin{aligned}
&H_{\boldsymbol{\theta}} P(\boldsymbol{\beta}, \boldsymbol{\gamma}, \boldsymbol{\theta}) \\
&= \sum_{i=1}^{n} \Bigg\{ - \delta_i \left[\frac{1}{\{\lambda_0(\kappa_i(y_i))\}^2} \right] \boldsymbol{\psi}(\kappa_i(y_i)) \boldsymbol{\psi}(\kappa_i(y_i))^T - \delta_i^L \boldsymbol{\Psi}(\kappa_i(y_i^R)) \left(\frac{\exp\{-\Lambda_0(\kappa_i(y_i^R))\}}{[1 - \exp\{-\Lambda_0(\kappa_i(y_i^R))\}]^2} \right) \boldsymbol{\Psi}(\kappa_i(y_i^R))^T \\
&\quad + \delta_i^I \Bigg(\left[\frac{1}{\exp\{-\Lambda_0(\kappa_i(y_i^L))\} - \exp\{-\Lambda_0(\kappa_i(y_i^R))\}} \right] \Big[\exp\{-\Lambda_0(\kappa_i(y_i^L))\} \boldsymbol{\Psi}(\kappa_i(y_i^L)) \boldsymbol{\Psi}(\kappa_i(y_i^L))^T \\
&\quad - \exp\{-\Lambda_0(\kappa_i(y_i^R))\} \boldsymbol{\Psi}(\kappa_i(y_i^R)) \boldsymbol{\Psi}(\kappa_i(y_i^R))^T \Big] - \left(\frac{1}{\left[\exp\{-\Lambda_0(\kappa_i(y_i^L))\} - \exp\{-\Lambda_0(\kappa_i(y_i^R))\}\right]^2} \right) \\
&\quad \times \left[- \exp\{-\Lambda_0(\kappa_i(y_i^L))\} \boldsymbol{\Psi}(\kappa_i(y_i^L)) + \exp\{-\Lambda_0(\kappa_i(y_i^R))\} \boldsymbol{\Psi}(\kappa_i(y_i^R)) \right] \\
&\quad \times \left[- \exp\{-\Lambda_0(\kappa_i(y_i^L))\} \boldsymbol{\Psi}(\kappa_i(y_i^L)) + \exp\{-\Lambda_0(\kappa_i(y_i^R))\} \boldsymbol{\Psi}(\kappa_i(y_i^R)) \right]^T \Bigg) \Bigg\} - 2h\boldsymbol{R}.
\end{aligned}$$

B.4.3 Expression for S_θ

From $\nabla_{\boldsymbol{\theta}} P(\boldsymbol{\beta}, \boldsymbol{\gamma}, \boldsymbol{\theta})$, $S_{\boldsymbol{\theta}}$ can be calculated and shown to be a diagonal matrix with elements

$$\frac{\theta_u}{\sum_{i=1}^n \left[\delta_i \Psi_u(\kappa_i(y_i)) + \delta_i^R \Psi_u(\kappa_i(y_i^L)) + \delta_i^I \left\{\frac{\Psi_u(\kappa_i(y_i^L))\exp(-\Lambda_0(\kappa_i(y_i^L)))}{\exp(-\Lambda_0(\kappa_i(y_i^L))) - \exp(-\Lambda_0(\kappa_i(y_i^R)))}\right\}\right] + 2h[\boldsymbol{R}_u \boldsymbol{\theta}]^+},$$

where $\boldsymbol{R}_u$ denotes the u-th row of matrix $\boldsymbol{R}$ and $[\boldsymbol{\eta}]^+ = \max\{0, \boldsymbol{\eta}\}$.

B.5 Second derivative with respect to $\boldsymbol{\beta}$ and $\boldsymbol{\gamma}$

The second derivative of $P(\boldsymbol{\beta}, \boldsymbol{\gamma}, \boldsymbol{\theta})$ with respect to $\boldsymbol{\beta}$ and $\boldsymbol{\gamma}$ is,

$$D_{\boldsymbol{\gamma}}\left(D_{\boldsymbol{\beta}} P(\boldsymbol{\beta}, \boldsymbol{\gamma}, \boldsymbol{\theta})^T\right),$$

which can be expressed as,

$$\begin{aligned}
&D_{\boldsymbol{\gamma}}\left(D_{\boldsymbol{\beta}} P(\boldsymbol{\beta}, \boldsymbol{\gamma}, \boldsymbol{\theta})^T\right) \\
&= \sum_{i=1}^n \boldsymbol{X}_i^T \Bigg\{\delta_i \Bigg[-\frac{\left\{\lambda_0(\kappa_i(y_i))\lambda_0^{'}(\kappa_i(y_i)) + \lambda_0(\kappa_i(y_i))\lambda_0^{''}(\kappa_i(y_i))\kappa_i(y_i) - (\lambda_0^{'}(\kappa_i(y_i)))^2\kappa_i(y_i)\right\}}{\{\lambda_0(\kappa_i(y_i))\}^2} \\
&\quad + \{\lambda_0(\kappa_i(y_i)) + \lambda_0^{'}(\kappa_i(y_i))\kappa_i(y_i)\}\Bigg] D_{\boldsymbol{\gamma}}\kappa_i(y_i)\Bigg\} + \delta_i^R\left\{\lambda_0^{'}(\kappa_i(y_i^L))\kappa_i(y_i^L) + \lambda_0(\kappa_i(y_i^L))\right\} D_{\boldsymbol{\gamma}}\kappa_i(y_i^L) \\
&\quad - \delta_i^L\Bigg(\frac{\exp\{-\Lambda_0(\kappa_i(y_i^R))\}\left\{\lambda_0(\kappa_i(y_i^R)) + \lambda_0^{'}(\kappa_i(y_i^R))\kappa_i(y_i^R)\right\} D_{\boldsymbol{\gamma}}\kappa_i(y_i^R)}{1 - \exp\{-\Lambda_0(\kappa_i(y_i^R))\}} \\
&\quad - \frac{\{\lambda_0(\kappa_i(y_i^R))\}^2\kappa_i(y_i^R)\exp\{\Lambda_0(\kappa_i(y_i^R))\}D_{\boldsymbol{\gamma}}\kappa_i(y_i^R)}{[1 - \exp\{-\Lambda_0(\kappa_i(y_i^R))\}]^2}\Bigg) \\
&\quad + \delta_i^I\Bigg[\Bigg\{\frac{\exp\{-\Lambda_0(\kappa_i(y_i^L))\}\left(\kappa_i(y_i^L)[\lambda_0^{'}(\kappa_i(y_i^L)) - \{\lambda_0(\kappa_i(y_i^L))\}^2] + \lambda_0(\kappa_i(y_i^L))\right)}{\exp\{-\Lambda_0(\kappa_i(y_i^L))\} - \exp\{-\Lambda_0(\kappa_i(y_i^R))\}}\Bigg\} D_{\boldsymbol{\gamma}}\kappa_i(y_i^L) \\
&\quad - \Bigg\{\frac{\exp\{-\Lambda_0(\kappa_i(y_i^R))\}\left(\kappa_i(y_i^R)[\lambda_0^{'}(\kappa_i(y_i^R)) - \{\lambda_0(\kappa_i(y_i^R))\}^2] + \lambda_0(\kappa_i(y_i^R))\right)}{\exp\{-\Lambda_0(\kappa_i(y_i^L))\} - \exp\{-\Lambda_0(\kappa_i(y_i^R))\}}\Bigg\} D_{\boldsymbol{\gamma}}\kappa_i(y_i^R) \\
&\quad + \frac{[\lambda_0(\kappa_i(y_i^R))\exp\{-\Lambda_0(\kappa_i(y_i^R))\}D_{\boldsymbol{\gamma}}\kappa_i(y_i^R) - \lambda_0(\kappa_i(y_i^L))\exp\{-\Lambda_0(\kappa_i(y_i^L))\}D_{\boldsymbol{\gamma}}\kappa_i(y_i^L)]}{[\exp\{-\Lambda_0(\kappa_i(y_i^L))\} - \exp\{-\Lambda_0(\kappa_i(y_i^R))\}]^2} \\
&\quad \times [\lambda_0(\kappa_i(y_i^R))\exp\{-\Lambda_0(\kappa_i(y_i^R))\}\kappa_i(y_i^R) - \lambda_0(\kappa_i(y_i^L))\exp\{-\Lambda_0(\kappa_i(y_i^L))\}\kappa_i(y_i^L)]\Bigg]\Bigg\}.
\end{aligned}$$

B.6 Second derivative with respect to $\boldsymbol{\beta}$ and $\boldsymbol{\theta}$

Next, the second derivative of $P(\boldsymbol{\beta}, \boldsymbol{\gamma}, \boldsymbol{\theta})$ with respect to $\boldsymbol{\beta}$ and $\boldsymbol{\theta}$ is,

$$D_{\boldsymbol{\theta}}\left(D_{\boldsymbol{\beta}} P(\boldsymbol{\beta}, \boldsymbol{\gamma}, \boldsymbol{\theta})^T\right),$$

which can be derived as,

$$
\begin{aligned}
&D_{\boldsymbol{\theta}}\left(D_{\boldsymbol{\beta}}P(\boldsymbol{\beta},\boldsymbol{\gamma},\boldsymbol{\theta})^T\right)\\
&=\sum_{i=1}^{n}\boldsymbol{X}_i\Bigg\{-\delta_i\left[\frac{\lambda_0(\kappa_i(y_i))\psi^{'}(\kappa_i(y_i))-\lambda_0^{'}(\kappa_i(y_i))\psi(\kappa_i(y_i))}{\{\lambda_0(\kappa_i(y_i))\}^2}-\psi(\kappa_i(y_i))\right]\kappa_i(y_i)+\delta_i^R\kappa_i(y_i^L)\psi(\kappa_i(y_i^L))\\
&\quad-\delta_i^L\left(\frac{\exp\{-\Lambda_0(\kappa_i(y_i^R))\}\{\psi(\kappa_i(y_i^R))-\Psi(\kappa_i(y_i^R))\lambda_0(\kappa_i(y_i^R))\}}{1-\exp\{-\Lambda_0(\kappa_i(y_i^R))\}}-\frac{[\exp\{-\Lambda_0(\kappa_i(y_i^R))\}]^2\lambda_0(\kappa_i(y_i^R))\Psi(\kappa_i(y_i^R))}{[1-\exp\{-\Lambda_0(\kappa_i(y_i^R))\}]^2}\right)\kappa_i(y_i^R)\\
&\quad+\delta_i^I\Bigg(\frac{\exp\{-\Lambda_0(\kappa_i(y_i^L))\}\{\psi(\kappa_i(y_i^L))-\Psi(\kappa_i(y_i^L))\lambda_0(\kappa_i(y_i^L))\}\kappa_i(y_i^L)}{\exp\{-\Lambda_0(\kappa_i(y_i^L))\}-\exp\{-\Lambda_0(\kappa_i(y_i^R))\}}\\
&\qquad-\frac{\exp\{-\Lambda_0(\kappa_i(y_i^R))\}\{\psi(\kappa_i(y_i^R))\Psi(\kappa_i(y_i^R))\lambda_0(\kappa_i(y_i^R))\}\kappa_i(y_i^R)}{\exp\{-\Lambda_0(\kappa_i(y_i^L))\}-\exp\{-\Lambda_0(\kappa_i(y_i^R))\}}\\
&\qquad+\frac{[\lambda_0(\kappa_i(y_i^R))\exp\{-\Lambda_0(\kappa_i(y_i^R))\}\kappa_i(y_i^R)-\lambda_0(\kappa_i(y_i^L))\exp\{-\Lambda_0(\kappa_i(y_i^L))\}\kappa_i(y_i^L)]}{[\exp\{-\Lambda_0(\kappa_i(y_i^L))\}-\exp\{-\Lambda_0(\kappa_i(y_i^R))\}]^2}\\
&\qquad\times[\Psi(\kappa_i(y_i^R))\exp\{-\Lambda_0(\kappa_i(y_i^R))\}-\Psi(\kappa_i(y_i^L))\exp\{-\Lambda_0(\kappa_i(y_i^L))\}]\Bigg)\Bigg\}.
\end{aligned}
$$

B.7 Second derivative with respect to γ and θ

Finally, the second derivative of $P(\boldsymbol{\beta},\boldsymbol{\gamma},\boldsymbol{\theta})$ with respect to $\boldsymbol{\gamma}$ and $\boldsymbol{\theta}$ is,

$$
D_{\boldsymbol{\theta}}\left(D_{\boldsymbol{\gamma}}P(\boldsymbol{\beta},\boldsymbol{\gamma},\boldsymbol{\theta})^T\right),
$$

which can be derived as,

$$
\begin{aligned}
&D_{\boldsymbol{\theta}}\left(D_{\boldsymbol{\gamma}}P(\boldsymbol{\beta},\boldsymbol{\gamma},\boldsymbol{\theta})^T\right)\\
&=\sum_{i=1}^{n}\Bigg\{\delta_i D_{\boldsymbol{\gamma}}\kappa_i(y_i)^T\left[\frac{\lambda_0(\kappa_i(y_i))\psi^{'}(\kappa_i(y_i))-\lambda_0^{'}(\kappa_i(y_i))\psi(\kappa_i(y_i))}{\{\lambda_0(\kappa_i(y_i))\}^2}-\psi(\kappa_i(y_i))\right]-\delta_i^R D_{\boldsymbol{\gamma}}\kappa_i(y_i^L)^T\psi(\kappa_i(y_i^L))\\
&\quad+\delta_i^L D_{\boldsymbol{\gamma}}\kappa_i(y_i^R)^T\Big(\frac{\exp\{-\Lambda_0(\kappa_i(y_i^R))\}\{\psi(\kappa_i(y_i^R))-\Psi(\kappa_i(y_i^R))\lambda_0(\kappa_i(y_i^R))\}}{1-\exp\{-\Lambda_0(\kappa_i(y_i^R))\}}\\
&\qquad-\frac{[\exp\{-\Lambda_0(\kappa_i(y_i^R))\}]^2\lambda_0(\kappa_i(y_i^R))\Psi(\kappa_i(y_i^R))}{[1-\exp\{-\Lambda_0(\kappa_i(y_i^R))\}]^2}\Big)\\
&\quad+\delta_i^I\Big(\frac{\exp\{\Lambda_0(\kappa_i(y_i^R))\}\{\psi(\kappa_i(y_i^R))-\Psi(\kappa_i(y_i^R))\lambda_0(\kappa_i(y_i^R))\}D_{\boldsymbol{\gamma}}\kappa_i(y_i^R)^T}{\exp\{-\Lambda_0(\kappa_i(y_i^L))\}-\exp\{-\Lambda_0(\kappa_i(y_i^R))\}}\\
&\qquad-\frac{\exp\{\Lambda_0(\kappa_i(y_i^L))\}\{\psi(\kappa_i(y_i^L))-\Psi(\kappa_i(y_i^L))\lambda_0(\kappa_i(y_i^L))\}D_{\boldsymbol{\gamma}}\kappa_i(y_i^L)^T}{\exp\{-\Lambda_0(\kappa_i(y_i^L))\}-\exp\{-\Lambda_0(\kappa_i(y_i^R))\}}\\
&\qquad+\frac{[\lambda_0(\kappa_i(y_i^L))\exp\{-\Lambda_0(\kappa_i(y_i^L))\}D_{\boldsymbol{\gamma}}\kappa_i(y_i^L)^T-\lambda_0(\kappa_i(y_i^R))\exp\{-\Lambda_0(\kappa_i(y_i^R))\}D_{\boldsymbol{\gamma}}\kappa_i(y_i^R)^T]}{[\exp\{-\Lambda_0(\kappa_i(y_i^L))\}-\exp\{-\Lambda_0(\kappa_i(y_i^R))\}]^2}\\
&\qquad\times[\Psi(\kappa_i(y_i^R))\exp\{-\Lambda_0(\kappa_i(y_i^R))\}-\Psi(\kappa_i(y_i^L))\exp\{-\Lambda_0(\kappa_i(y_i^L))\}]\Big)\Bigg\}.
\end{aligned}
$$

C Technical connection of Section 4.4 to Ma et al. (2021) and Ma (2024)

The asymptotic results stated in Section 4.4 of the main paper follow the same general strategy as Ma et al. (2021) for partly interval-censored survival data and Ma (2024, Chapter 4) for semiparametric AFT models with partly interval-censored failure times and time-varying covariates. We briefly summarise the connection here.

Let

$$\boldsymbol{\eta} = (\boldsymbol{\theta}^T, \boldsymbol{\beta}^T, \boldsymbol{\gamma}^T)^T,$$

where θ denotes the basis coefficients in the approximation

$$\lambda_0(\kappa) = \sum_{u=1}^{m} \theta_u \psi_u(\kappa), \qquad \theta_u \geq 0,\ u = 1, \ldots, m.$$

The penalised log-likelihood can be written as

$$P(\boldsymbol{\eta}) = \ell(\boldsymbol{\eta}) - hJ(\boldsymbol{\theta}),$$

where $J(\boldsymbol{\theta}) = \boldsymbol{\theta}^T \boldsymbol{R} \boldsymbol{\theta}$ for the roughness penalty considered in this paper.

The argument for consistency uses the same sieve logic as Ma et al. (2021): the baseline hazard is approximated by a finite basis expansion, with $m \to \infty$, $m/n \to 0$, and $h/n \to 0$. Under boundedness of the covariate processes and basis functions, boundedness of the penalty, and adequacy of the basis approximation, the induced parameter space is sufficiently regular for the maximiser of the penalised log-likelihood to converge to the true parameter. Relative to Ma et al. (2021), the main modification is that the baseline hazard and survival functions are evaluated at the transformed time scale

$$\kappa_i(t; \boldsymbol{\beta}, \boldsymbol{\gamma}) = e^{-\boldsymbol{x}_i \boldsymbol{\beta}} \int_0^t e^{-\boldsymbol{z}_i(s) \boldsymbol{\gamma}} ds,$$

rather than at the observed time t. The required derivatives of κ_i, λ_0, and S_0 with respect to $\boldsymbol{\beta}$, γ, and $\boldsymbol{\theta}$ are listed in Section B of this supplementary material, which ensures that the score and Hessian quantities used in the asymptotic expansion are well defined.

For large-sample approximate normality, we adopt the same fixed-m strategy as Ma et al. (2021) and Ma (2024). With m fixed, $\boldsymbol{\eta}$ is finite-dimensional, and the Karush–Kuhn–Tucker conditions imply that the constrained maximiser $\hat{\boldsymbol{\eta}}$ satisfies the reduced score equation

$$\boldsymbol{U}^T \frac{\partial P(\hat{\boldsymbol{\eta}})}{\partial \boldsymbol{\eta}} = 0,$$

where $\boldsymbol{U}$ removes the coordinates associated with active constraints among $\boldsymbol{\theta} \geq 0$. A

first-order Taylor expansion of the reduced score around the true parameter $\boldsymbol{\eta}_0$, together with the law of large numbers and central limit theorem, yields the asymptotic normal approximation stated in Section 4.4.

The role of $\boldsymbol{U}$ in this paper is identical to that in Ma et al. (2021): if some of the θ_u are zero at the solution and their corresponding gradient components are negative, then these constraints are active and must be excluded from the unconstrained information calculation. This yields the reduced covariance structure

$$\tilde{\boldsymbol{G}}(\boldsymbol{\eta})^{-1} = \boldsymbol{U}\{\boldsymbol{U}^T F(\boldsymbol{\eta})\boldsymbol{U}\}^{-1}\boldsymbol{U}^T,$$

and, in finite samples with non-zero smoothing, the practical covariance estimator used in Section 4.4 is obtained by replacing the limiting information with the observed negative Hessian and adding the penalty term $h\boldsymbol{R}$.

D Simulation study under continuous time-varying covariate generation

To assess the performance of the proposed method when the time-varying covariate is continuous, we considered an additional simulation setting. The time-fixed covariates were generated as in the main simulation study, while the binary time-varying covariate was replaced by the subject-specific continuous trajectory (similar to Scenario 2 Condition 3 in Webb and Ma (2023))

$$z_i(t) = \zeta_i \sin(2t), \qquad \zeta_i \sim N(1, 1)$$

This continuous covariate process was represented as piecewise constant over subject-specific observation intervals. For subject i, the initial number of observation intervals was generated as $n_i = 1 + \text{Poisson}(\varrho)$, so that each subject had at least one interval, and the interval lengths were drawn independently from $\text{Unif}(\tau_{\min}, \tau_{\max})$. Let t_{ia} denote the left endpoint of the a-th interval for subject i. The time-varying covariate value on that interval was set to $z_{ia} = \zeta_i \sin(2t_{ia})$, and was carried forward until the next observation time. Thus, the continuous trajectory was approximated by a piecewise-constant covariate history, and the same history was used both for event-time generation and for model fitting.

Event times were generated under the AFT model by inverse transform sampling on the baseline scale. For each subject, a target value w_i was first obtained from the chosen baseline survival distribution. Here, we illustrate this using the same Weibull distribution as defined in the Simulation section of the main paper. Different from Webb and Ma (2023), the event time t_i was then determined by solving

$$e^{-\boldsymbol{x}_i \boldsymbol{\beta}} \int_0^{t_i} e^{-\boldsymbol{z}_i(s)\boldsymbol{\gamma}} ds = w_i.$$

Because $z_i(s)$ is constant on each generated observation interval, this integral was evaluated by accumulating contributions interval by interval. If the event-time target was not reached by the initially generated observation grid, additional observation intervals were generated using the same $\text{Unif}(\tau_{\min}, \tau_{\max})$ gap distribution until the event time was located. This extension step is necessary because the observation grid is generated before the event time is known.

In this simulation study, the true values of β_1 and β_2 were set to 1 and -1, respectively, while the true value of γ was set to -0.1. In addition, $\varrho = 10$, $\tau_{\min} = 0$, and $\tau_{\max} = 0.5$ were adopted to control the number of observation intervals and their lengths. Two sample sizes, $n = 100$ and $n = 1000$, were chosen to represent both small and large samples.

Table 1: Results obtained for $\hat{\beta}_1$, $\hat{\beta}_2$ and $\hat{\gamma}$ for varying sample sizes and different event proportions. These results are based on 200 repetitions.

	$n = 100,\ m = 5$			$n = 1000,\ m = 10$		
	$\pi^E = 0.3$			$\pi^E = 0.3$		
	$\hat{\beta}_1$	$\hat{\beta}_2$	$\hat{\gamma}$	$\hat{\beta}_1$	$\hat{\beta}_2$	$\hat{\gamma}$
Bias	0.0064	-0.0383	-0.0691	0.0056	0.0050	0.0017
MCSD	0.1069	0.0861	0.2150	0.0326	0.0209	0.0553
AASD	0.1180	0.0626	0.1786	0.0330	0.0199	0.0495
CP (MCSD)	0.945	0.925	0.930	0.940	0.935	0.945
CP (AASD)	0.980	0.835	0.890	0.940	0.930	0.910
	$\pi^E = 0.7$			$\pi^E = 0.7$		
	$\hat{\beta}_1$	$\hat{\beta}_2$	$\hat{\gamma}$	$\hat{\beta}_1$	$\hat{\beta}_2$	$\hat{\gamma}$
Bias	-0.0007	-0.0088	-0.0111	0.0020	0.0017	0.0019
MCSD	0.0752	0.0631	0.1368	0.0270	0.0203	0.0396
AASD	0.0803	0.0428	0.1105	0.0250	0.0150	0.0338
CP (MCSD)	0.955	0.935	0.945	0.945	0.945	0.960
CP (AASD)	0.965	0.800	0.900	0.930	0.840	0.895

These results demonstrate that, under the continuous time-varying covariate setting, the proposed approach achieves small biases across sample sizes. Furthermore, the coverage probabilities based on the MCSDs for the proposed method are close to the nominal 95%, indicating accurate uncertainty quantification.

For larger proportions of exact observations ($\pi^E = 0.7$), the AASD tends to underestimate the empirical variability, resulting in some undercoverage. This appears to be a finite-sample effect, likely due to the sharper likelihood induced by the increased number of exact event times. The discrepancy is reduced as the sample size increases, indicating that the asymptotic approximation improves in larger samples.

Figures 1 and 2 display the estimated baseline hazard and survival functions over a grid of κ values. Overall, the estimated curves closely follow the true baseline functions, indicating that the proposed method accurately recovers both the hazard and survival functions. The confidence intervals provide reasonable coverage across the range of κ, with improved precision observed for larger sample sizes, reflecting the consistency of the estimator.

Overall, the results indicate that the proposed method remains accurate and stable when the covariate process is continuous rather than binary.

(a) $\pi^E = 0.3, n = 100, m = 5$

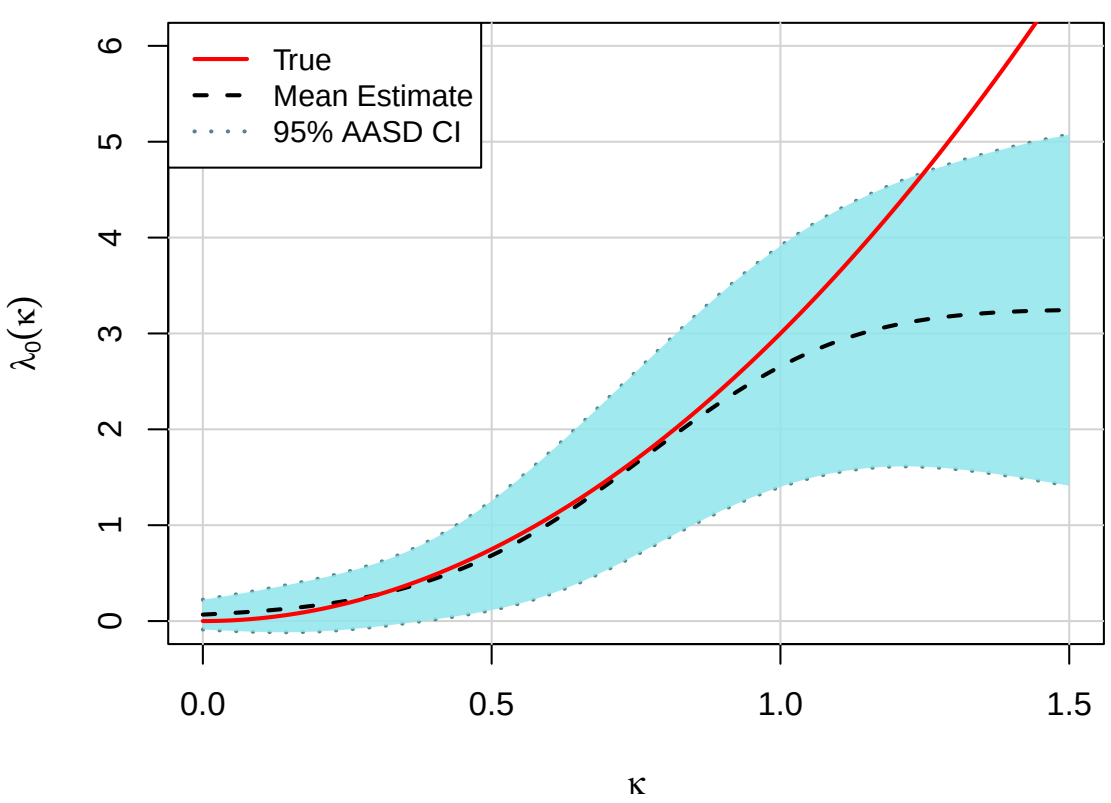

(b) $\pi^E = 0.7, n = 100, m = 5$

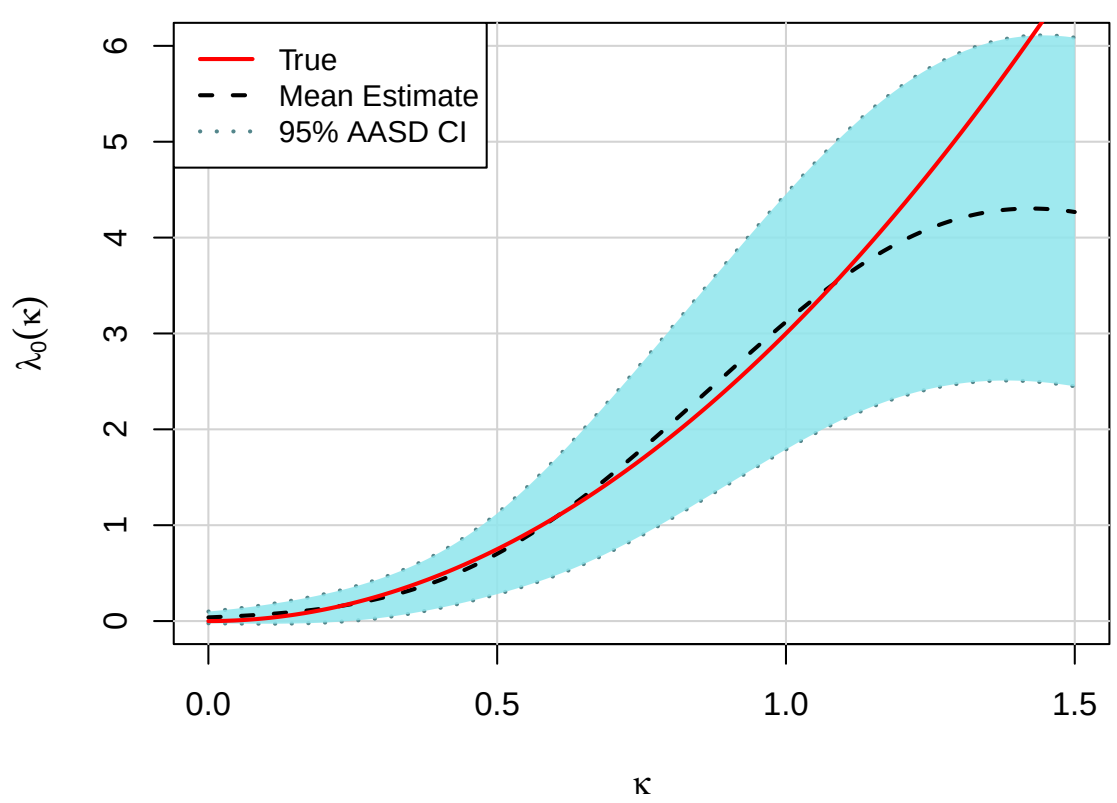

(c) $\pi^E = 0.3, n = 1000, m = 10$

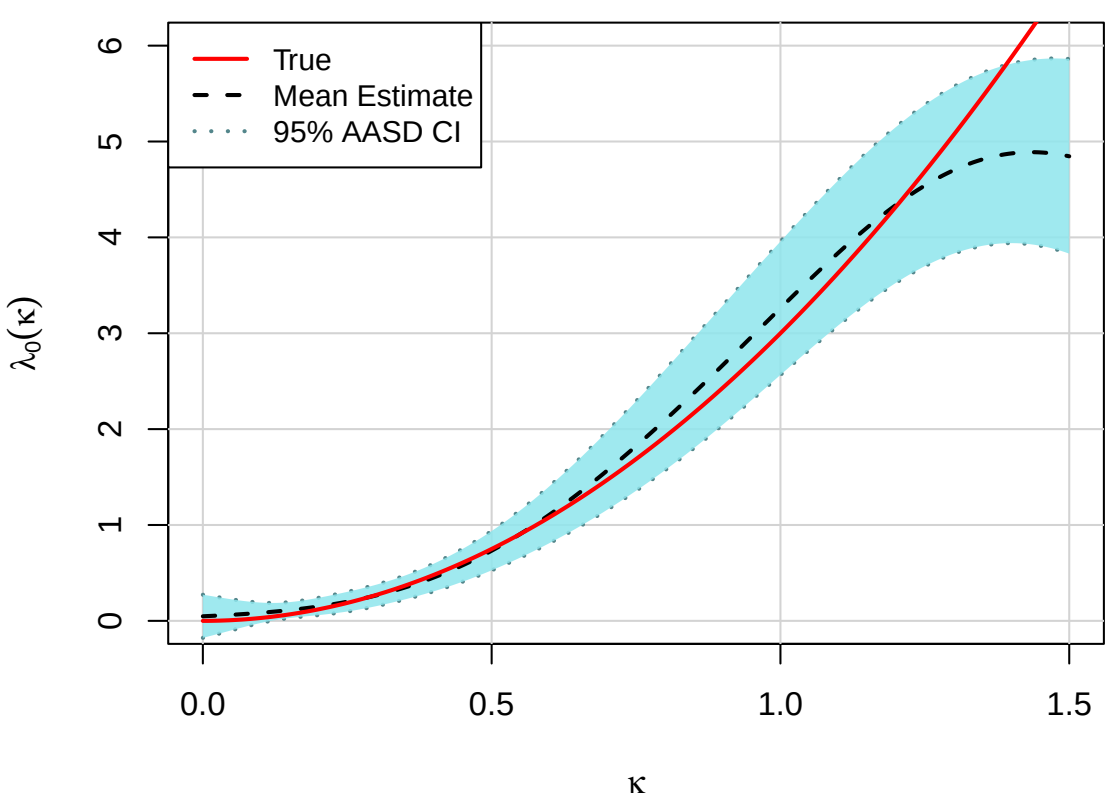

(d) $\pi^E = 0.7, n = 1000, m = 10$

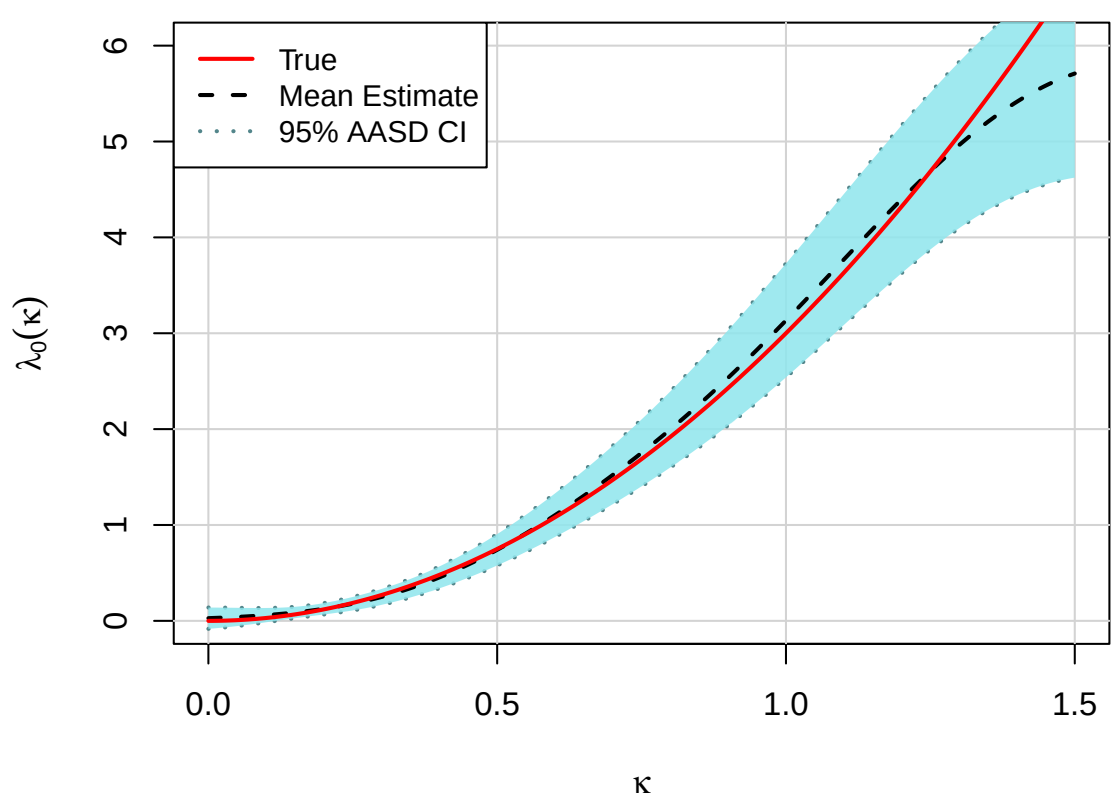

Figure 1: Figures of the weibull baseline hazard functions, in the continuous time-vary covariate setting, on a grid of κ values. Each plot includes the true baseline hazard curve (solid red line), the mean estimated baseline hazard curve (dashed black line) and area bound by the 95% pointwise confidence interval generated using AASD (coloured region).

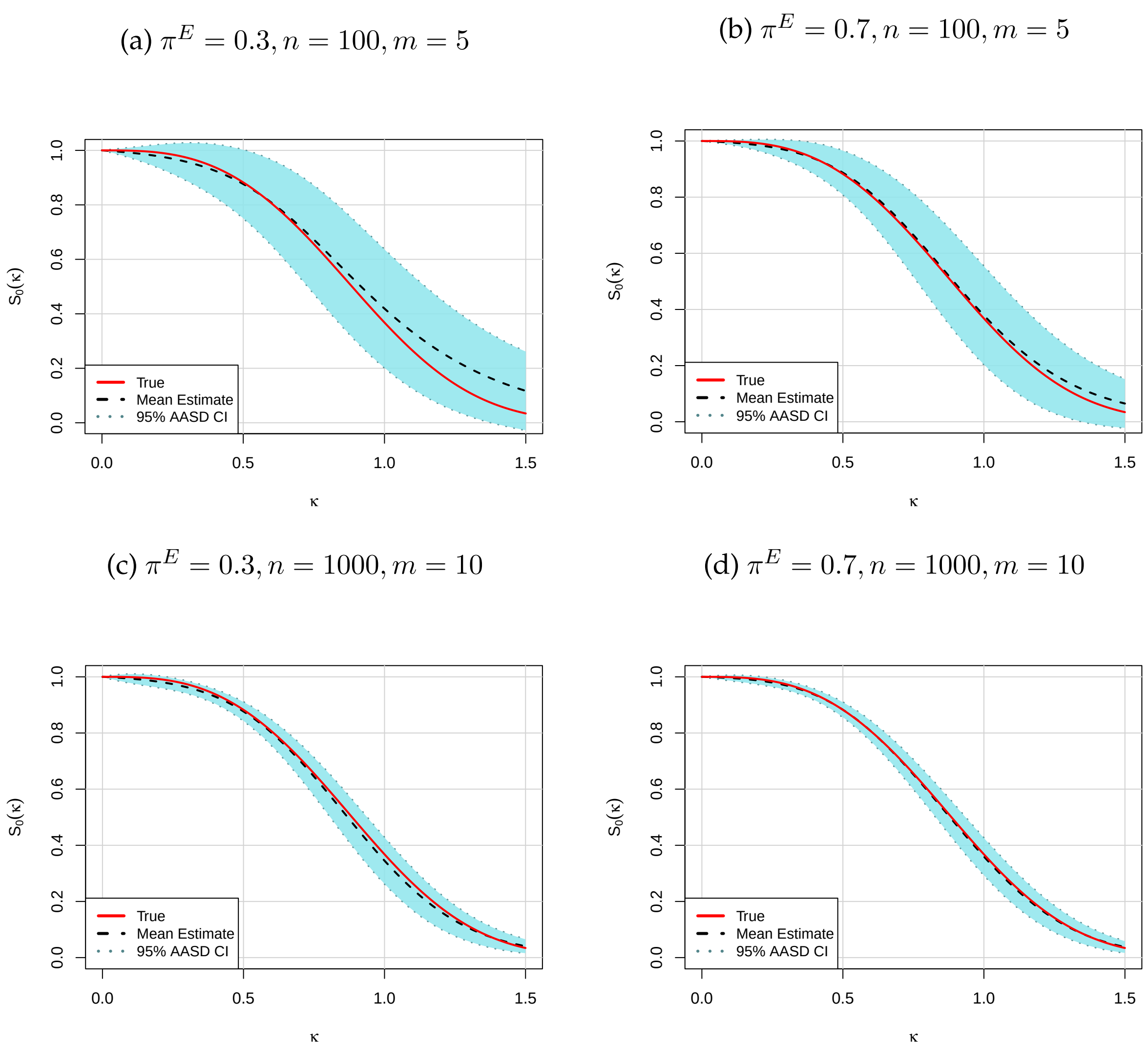

(a) $\pi^E = 0.3, n = 100, m = 5$

(b) $\pi^E = 0.7, n = 100, m = 5$

(c) $\pi^E = 0.3, n = 1000, m = 10$

(d) $\pi^E = 0.7, n = 1000, m = 10$

Figure 2: Figures of the weibull baseline survival functions, in the continuous time-vary covariate setting, on a grid of κ values. Each plot includes the true baseline survival curve (solid red line), the mean estimated baseline survival curve (dashed black line) and area bound by the 95% pointwise confidence interval generated using AASD (coloured region).

E Simulation results: proposed vs midpoint imputation approach

(a) $\pi^E = 0.3, n = 100, m = 5$

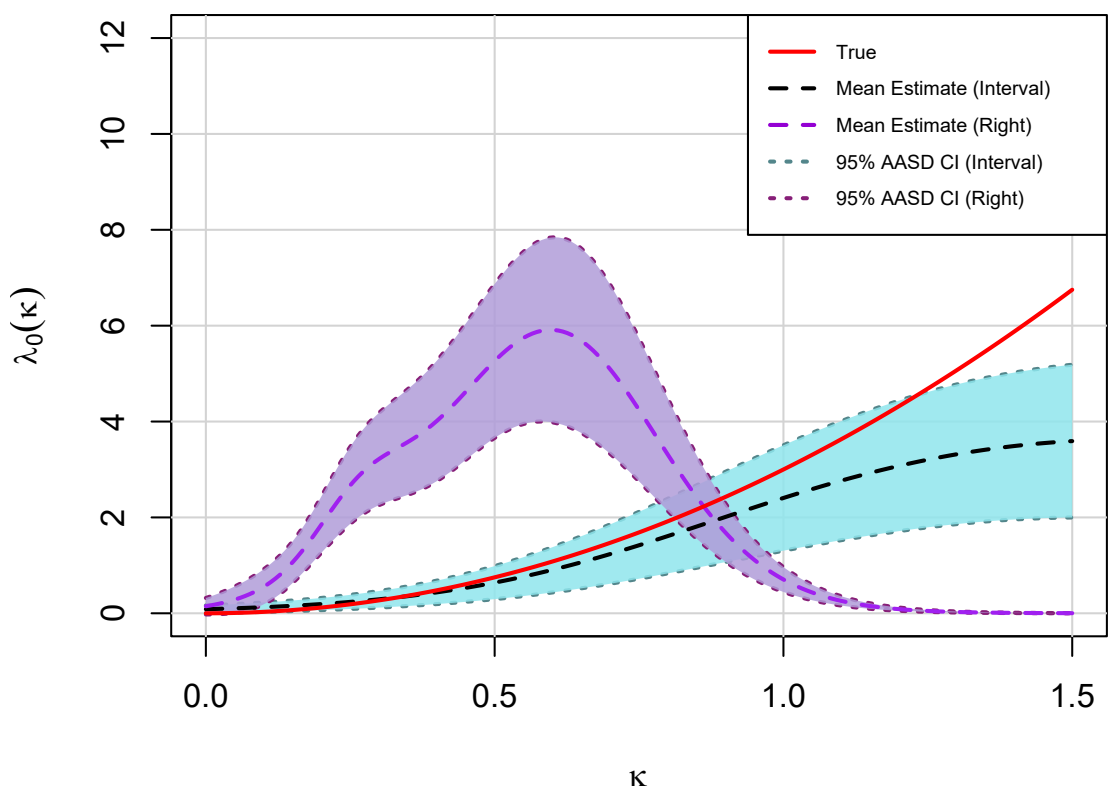

(b) $\pi^E = 0.7, n = 100, m = 5$

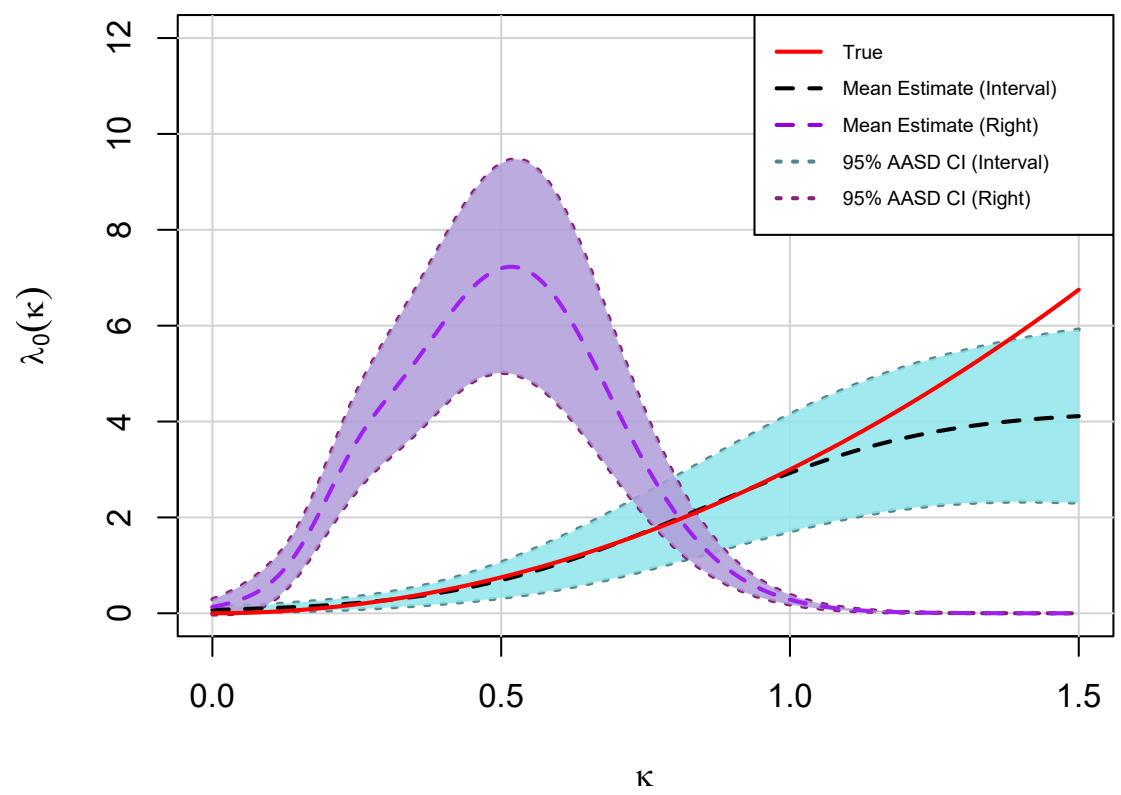

(c) $\pi^E = 0.3, n = 1000, m = 10$

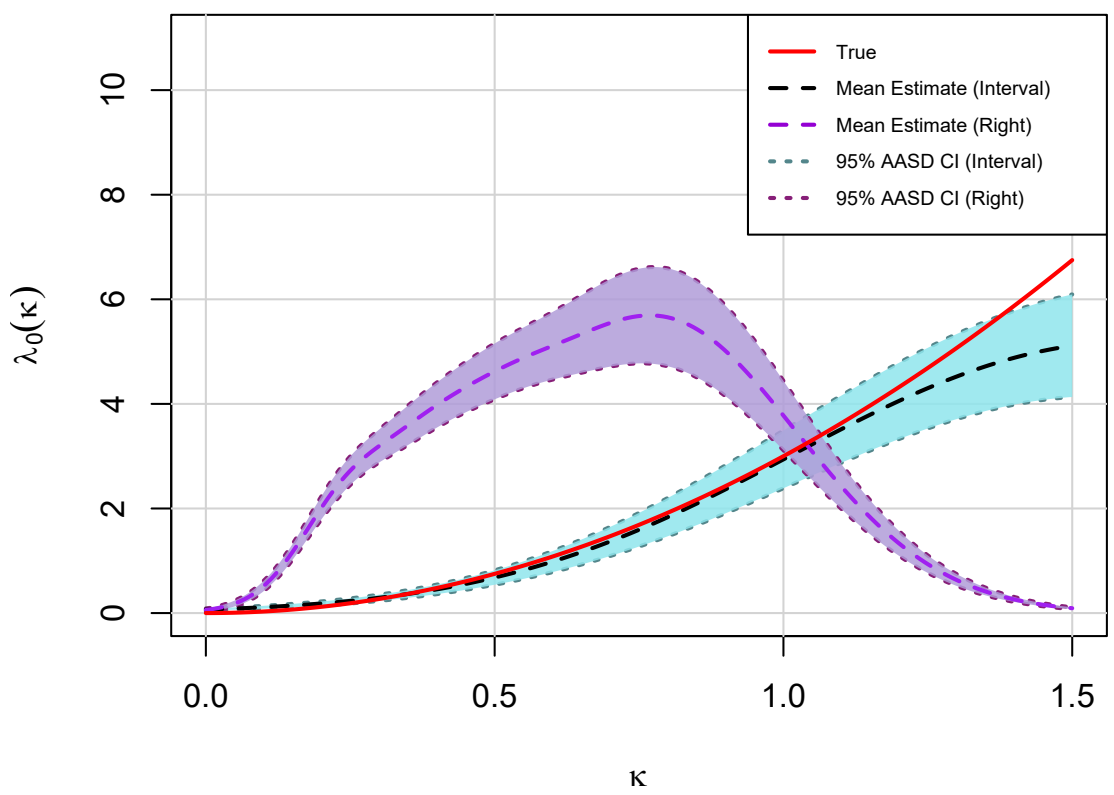

(d) $\pi^E = 0.7, n = 1000, m = 10$

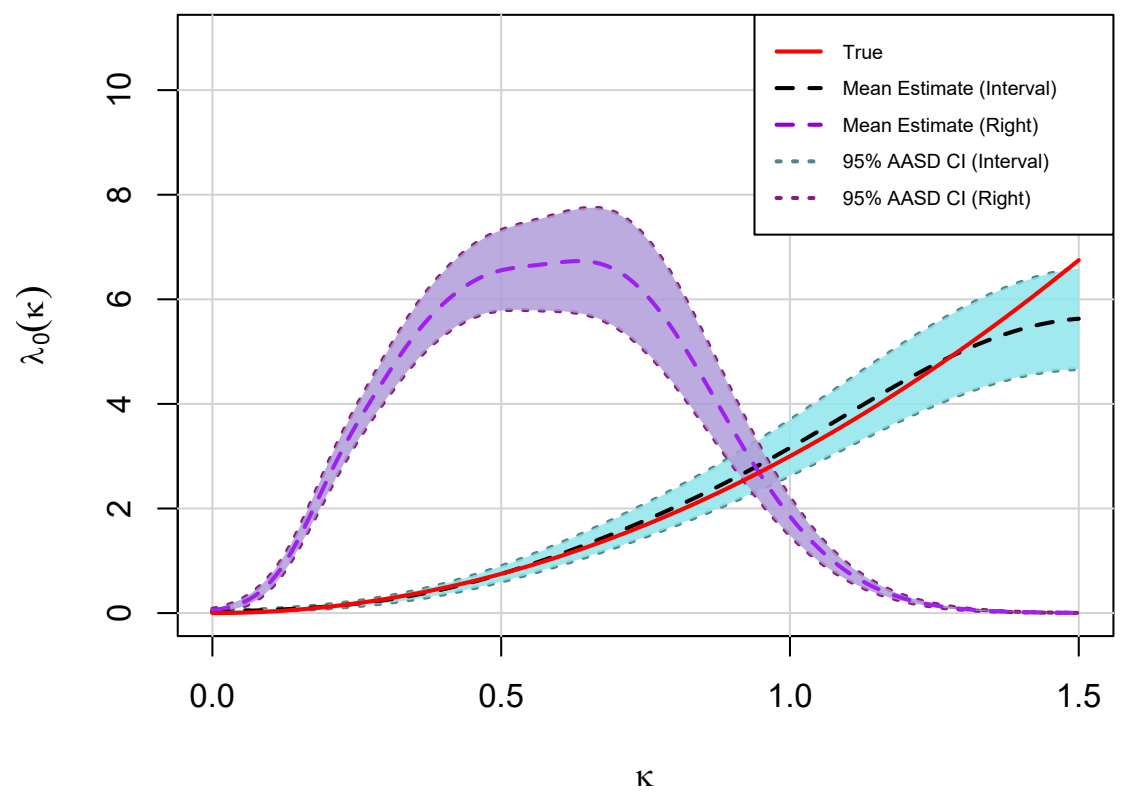

Figure 3: Figures of the baseline hazard functions on a grid of κ values. Each plot includes the true baseline hazard curve (solid red line), the mean estimated baseline hazard curves (dashed black line (proposed approach) and dashed purple line (midpoint imputation approach)) and area bound by the 95% pointwise confidence interval generated using AASD (coloured regions).

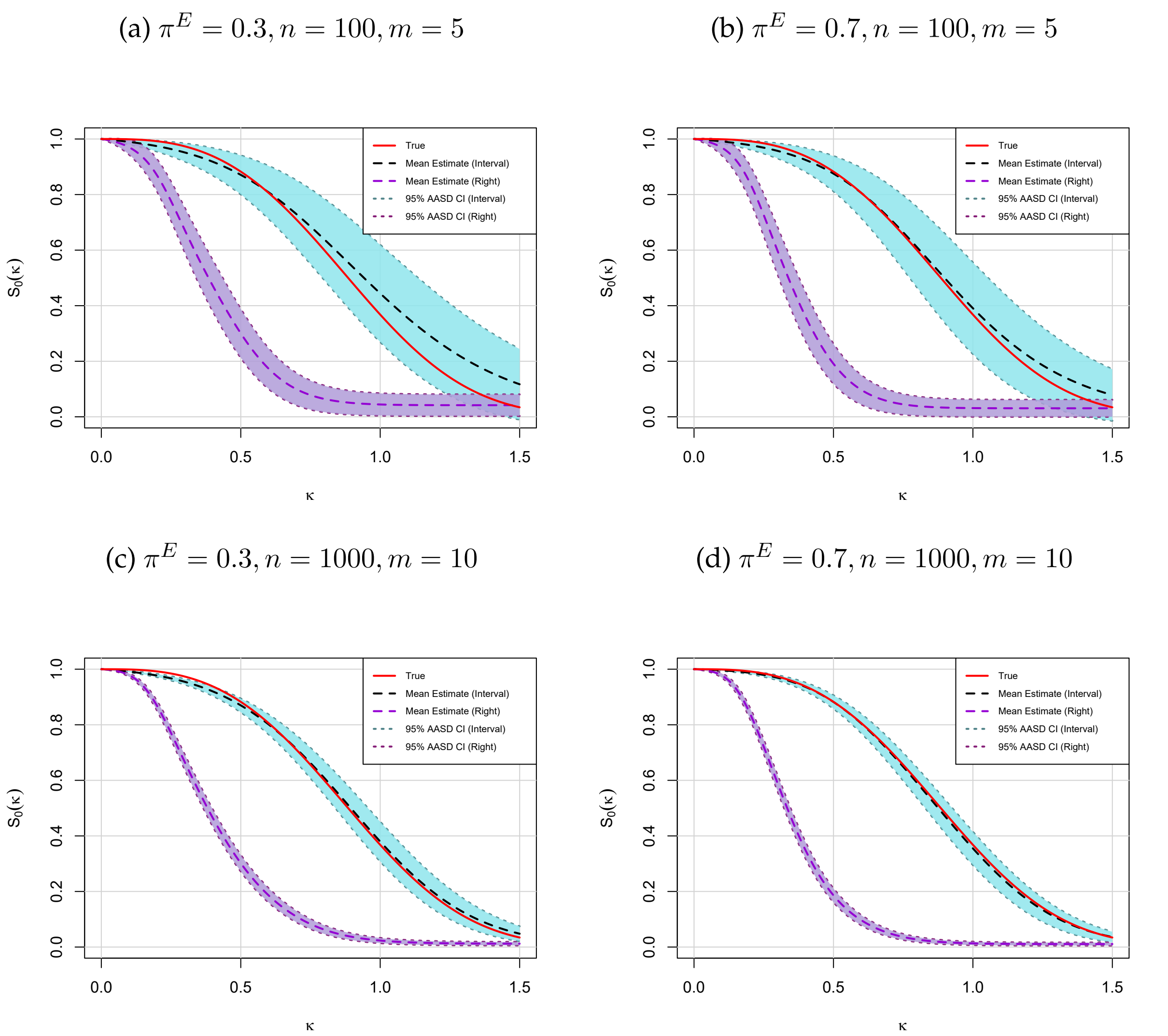

Figure 4: Figures of the baseline survival functions on a grid of κ values. Each plot includes the true baseline survival curve (solid red line), the mean estimated baseline survival curves (dashed black line (proposed approach) and dashed purple line (mid-point imputation approach)) and area bound by the 95% pointwise confidence interval generated using AASD (coloured regions).

F Additional simulation results: log-logistic setting

This section presents additional results for the simulation study under a log-logistic error distribution in the AFT model, corresponding to a non-monotone baseline hazard. The simulation settings were updated accordingly to reflect the log-logistic baseline specification with shape parameter $\alpha = 5$ and scale parameter $\psi = 1$.

The corresponding baseline hazard, specified by a log-logistic model with the above parameter values, is given by $\lambda_0(\kappa) = (5\kappa^4)/(1+\kappa^5)$, and the corresponding baseline survival function is $S_0(\kappa) = 1/(1+\kappa^5)$.

In this simulation, the true values of β_1 and β_2 were set to 1 and -0.3, respectively, while the true value of γ was set to -0.1. Two sample sizes, $n = 250$ and $n = 1000$, were chosen to represent both small and large samples. A slightly larger minimum sample size than in the Weibull setting was considered to ensure stable estimation under the more complex log-logistic baseline with non-monotone hazard.

Table 2: Results obtained for $\hat{\beta}_1$, $\hat{\beta}_2$ and $\hat{\gamma}$ for varying sample sizes and different event proportions. These results are based on 200 repetitions.

	$n = 250,\ m = 7$			$n = 1000,\ m = 10$		
	$\pi^E = 0.3$			$\pi^E = 0.3$		
	$\hat{\beta}_1$	$\hat{\beta}_2$	$\hat{\gamma}$	$\hat{\beta}_1$	$\hat{\beta}_2$	$\hat{\gamma}$
Bias	0.0016	-0.0069	-0.0621	0.0042	-0.0020	-0.0107
MCSD	0.0694	0.0333	0.1123	0.0353	0.0143	0.0505
AASD	0.0726	0.0327	0.0993	0.0344	0.0157	0.0489
CP (MCSD)	0.936	0.932	0.904	0.956	0.948	0.932
CP (AASD)	0.952	0.924	0.854	0.948	0.984	0.924
	$\pi^E = 0.7$			$\pi^E = 0.7$		
	$\hat{\beta}_1$	$\hat{\beta}_2$	$\hat{\gamma}$	$\hat{\beta}_1$	$\hat{\beta}_2$	$\hat{\gamma}$
Bias	-0.0080	-0.0042	-0.0485	-0.0008	-0.0005	-0.0048
MCSD	0.0591	0.0261	0.0959	0.0307	0.0122	0.0451
AASD	0.0588	0.0262	0.0816	0.0284	0.0128	0.0404
CP (MCSD)	0.968	0.932	0.916	0.956	0.952	0.944
CP (AASD)	0.968	0.932	0.868	0.940	0.964	0.892

Figures 5 and 6 display the estimated baseline hazard and survival functions over a grid of κ values. Overall, the estimated curves closely follow the true baseline functions, indicating that the proposed method accurately recovers both the hazard and survival functions under a non-monotone setting. The confidence intervals provide reasonable coverage across the range of κ. The agreement between the true and estimated curves is particularly strong for larger sample sizes, reflecting the consistency of the estimator.

These results support the robustness of the proposed approach to different baseline hazard shapes, extending beyond the Weibull setting in the main manuscript.

(a) $\pi^E = 0.3, n = 250, m = 7$

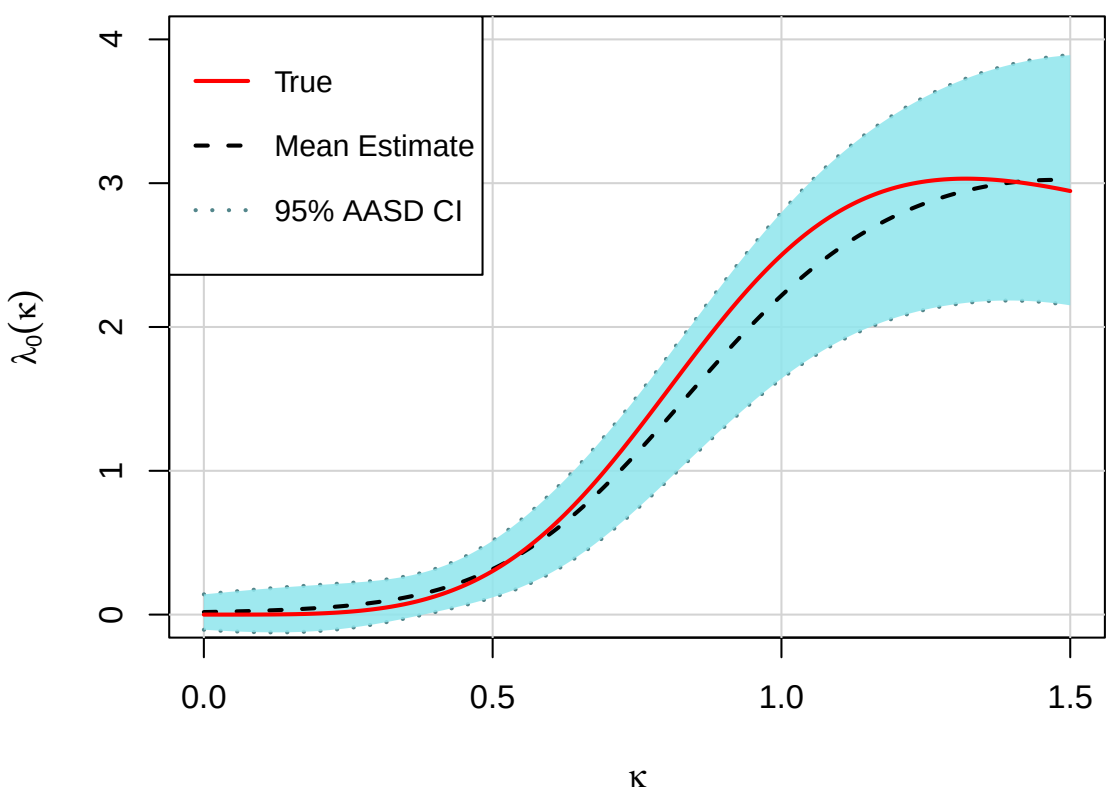

(b) $\pi^E = 0.7, n = 250, m = 7$

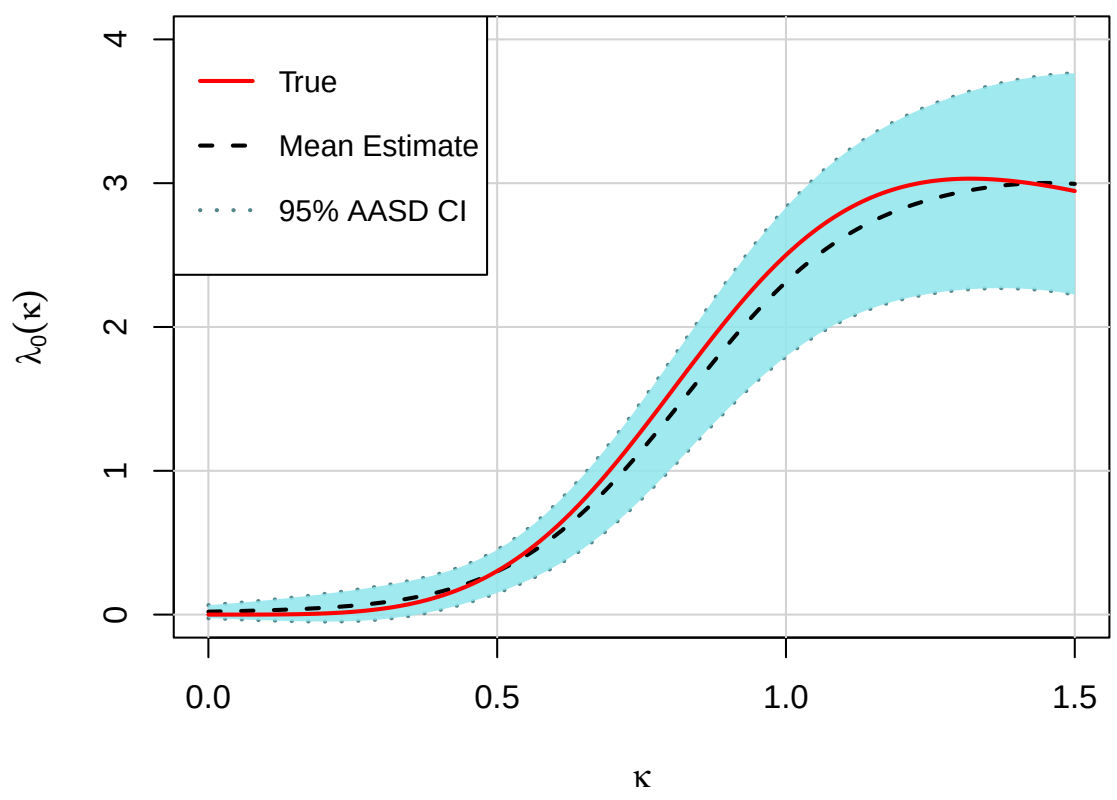

(c) $\pi^E = 0.3, n = 1000, m = 10$

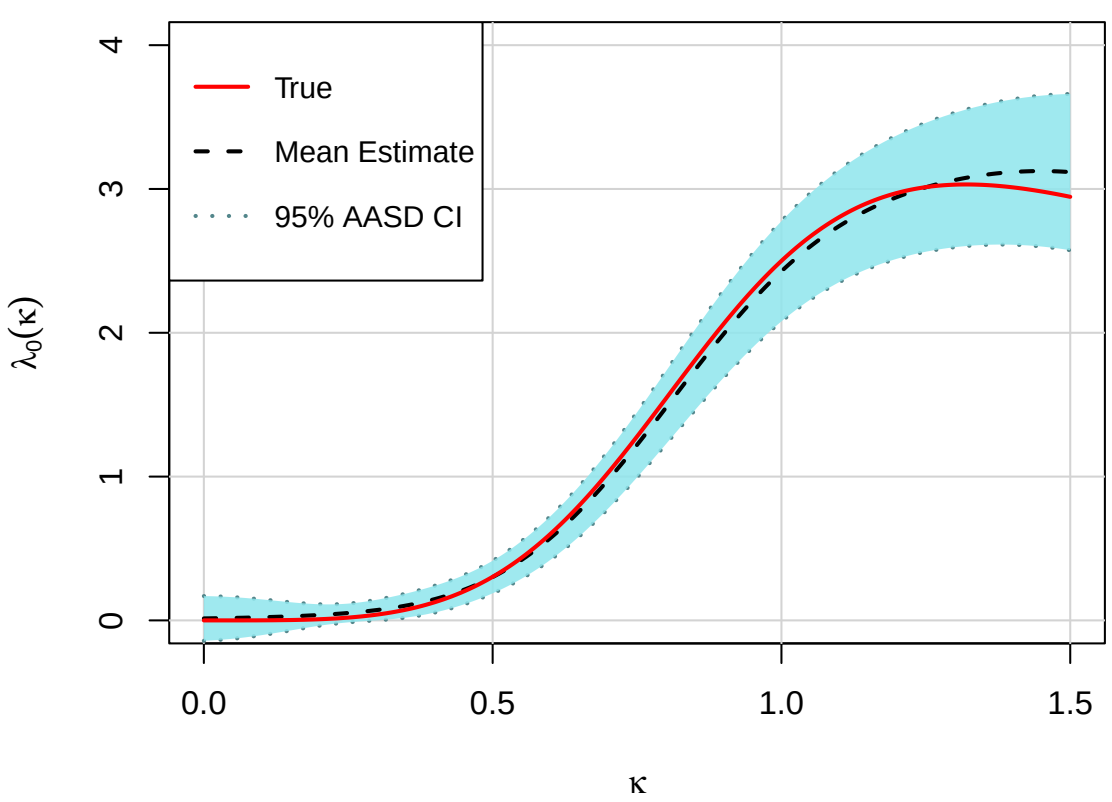

(d) $\pi^E = 0.7, n = 1000, m = 10$

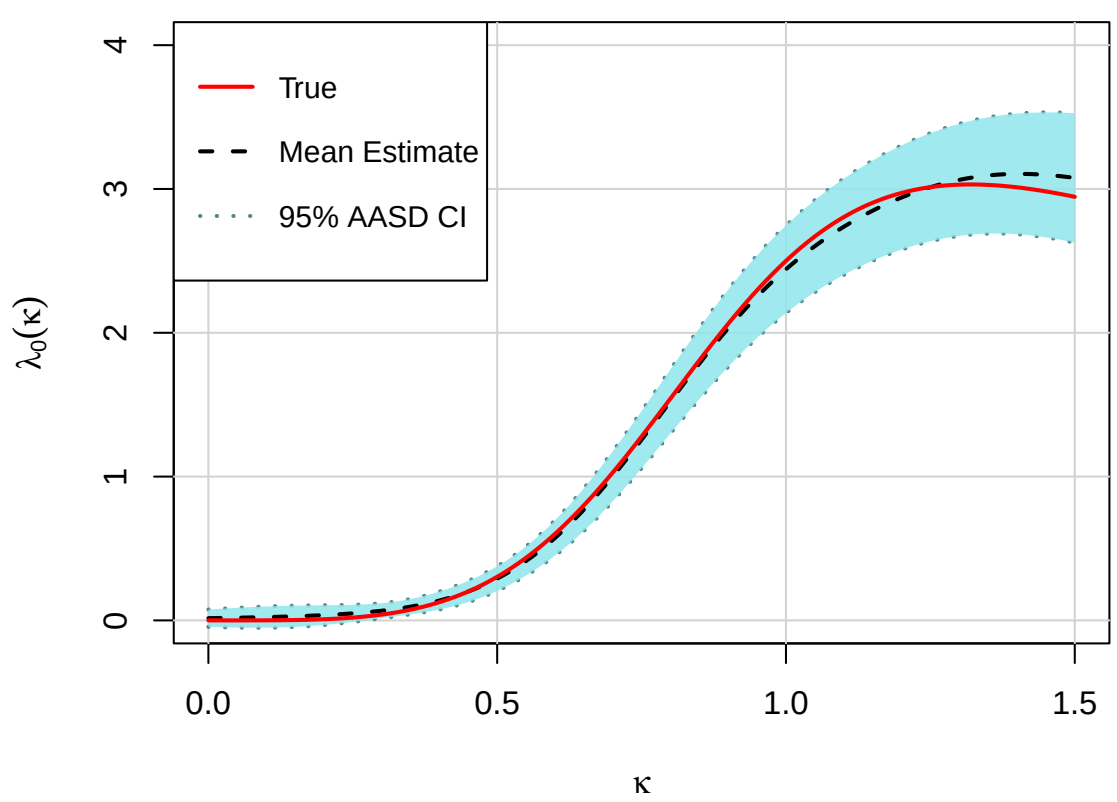

Figure 5: Figures of the log-logistic baseline hazard functions on a grid of κ values. Each plot includes the true baseline hazard curve (solid red line), the mean estimated baseline hazard curve (dashed black line) and area bound by the 95% pointwise confidence interval generated using AASD (coloured region).

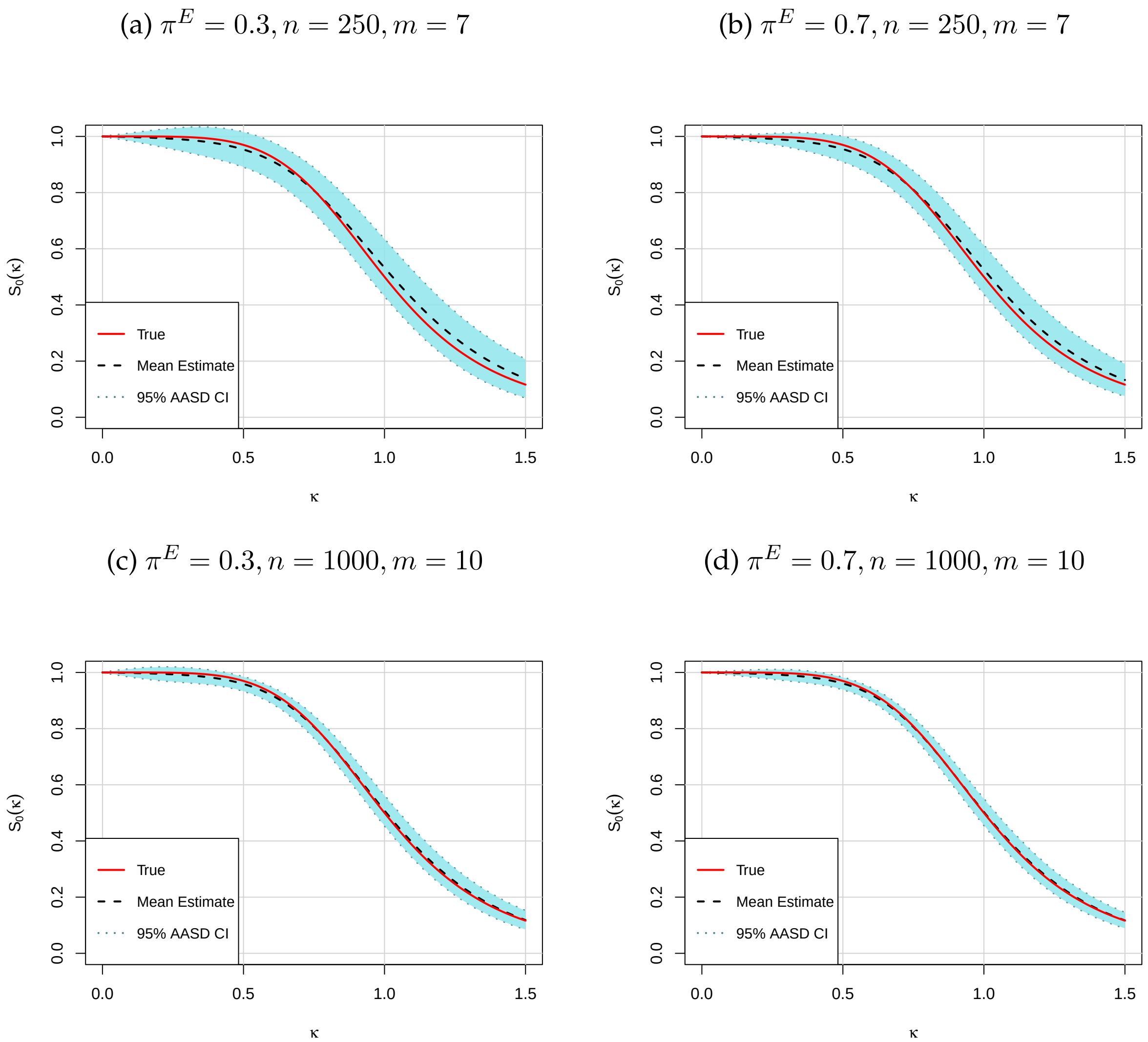

Figure 6: Figures of the log-logistic baseline survival functions on a grid of κ values. Each plot includes the true baseline survival curve (solid red line), the mean estimated baseline survival curve (dashed black line) and area bound by the 95% pointwise confidence interval generated using AASD (coloured region).
